# Transcriptomic Analysis of Inflammatory Cardiomyopathy Identifies Molecular Signatures of Disease and Informs *in silico* Prediction of a Network-Based Rationale for Therapy

**DOI:** 10.3389/fimmu.2021.640837

**Published:** 2021-03-05

**Authors:** Kamayani Singh, Hai Fang, Graham Davies, Benjamin Wright, Helen Lockstone, Richard O. Williams, Daniela Ciháková, Julian C. Knight, Shoumo Bhattacharya

**Affiliations:** ^1^RDM Cardiovascular Medicine, University of Oxford, Oxford, United Kingdom; ^2^Wellcome Centre for Human Genetics, University of Oxford, Oxford, United Kingdom; ^3^Nuffield Department of Medicine, University of Oxford, Oxford, United Kingdom; ^4^Kennedy Institute of Rheumatology, University of Oxford, Oxford, United Kingdom; ^5^Division of Immunology, Department of Pathology, School of Medicine, Johns Hopkins University, Baltimore, MD, United States

**Keywords:** myocarditis, autoimmune, transcriptome, network, diagnosis, therapy

## Abstract

Inflammatory cardiomyopathy covers a group of diseases characterized by inflammation and dysfunction of the heart muscle. The immunosuppressive agents such as prednisolone, azathioprine and cyclosporine are modestly effective treatments, but a molecular rationale underpinning such therapy or the development of new therapeutic strategies is lacking. We aimed to develop a network-based approach to identify therapeutic targets for inflammatory cardiomyopathy from the evolving myocardial transcriptome in a mouse model of the disease. We performed bulk RNA sequencing of hearts at early, mid and late time points from mice with experimental autoimmune myocarditis. We identified a cascade of pathway-level events involving early activation of cytokine and chemokine-signaling pathways that precede leucocyte infiltration and are followed by innate immune, antigen-presentation, complement and cell-adhesion pathway activation. We integrated these pathway events into a network-like representation from which we further identified a 50-gene subnetwork that is predominantly induced during the course of autoimmune myocardial inflammation. We developed a combinatorial attack strategy where we quantify network tolerance to combinatorial node removal to determine target-specific therapeutic potential. We find that combinatorial attack of *Traf2, Nfkb1, Rac1*, and *Vav1* disconnects 80% of nodes from the largest network component. Two of these nodes, *Nfkb1* and *Rac1*, are directly targeted by prednisolone and azathioprine respectively, supporting the idea that the methodology developed here can identify valid therapeutic targets. Whereas *Nfkb1* and *Rac1* removal disconnects 56% of nodes, we show that additional removal of *Btk* and *Pik3cd* causes 72% node disconnection. In conclusion, transcriptome profiling, pathway integration, and network identification of autoimmune myocardial inflammation provide a molecular signature applicable to the diagnosis of inflammatory cardiomyopathy. Combinatorial attack provides a rationale for immunosuppressive therapy of inflammatory cardiomyopathy and provides an *in silico* prediction that the approved therapeutics, ibrutinib and idelalisib targeting *Btk* and *Pik3cd* respectively, could potentially be re-purposed as adjuncts to immunosuppression.

## Introduction

Inflammatory cardiomyopathy covers a group of diseases characterized by inflammation and dysfunction of the heart muscle, and often progresses to heart failure and death [reviewed in (1)]. Inflammatory cardiomyopathy can be triggered by infections (most commonly viral in the developed world), immune-mediated mechanisms and toxins (1, 2). Immunosuppressive therapy using agents such as prednisolone, cyclosporine and azathioprine is modestly effective in chronic virus-negative, giant cell and active autoimmune forms of inflammatory cardiomyopathy, and is recommended for such patients (2). A key challenge is to understand the molecular rationale underlying current immunosuppressive therapy for inflammatory cardiomyopathy, and to develop new therapeutic strategies that would improve patient outcomes.

Rodent models of autoimmune myocarditis have been extensively studied to understand the mechanisms of inflammatory cardiomyopathy [reviewed in (3)]. Cardiac inflammation, typically manifested by infiltration of myeloid and CD4^+^ T cells, occurs between 14 and 21 days after immunization, following which there is progressive fibrosis modeling clinical cardiomyopathy (3). A number of genes have been shown to be necessary for the development of inflammatory cardiomyopathy in this model and include the TLR-signaling adaptor *Myd88* (4), cytokines *Tnf* (5), *Il23a* (6), *Csf1* (7), *Csf2* (8), *Il6* (9), *Il1r1* (9), *Il17a* (10), and *Il4* (11), chemokines and their receptors *Ccl2* (12), *Ccl3* (12), *Ccr1* (13), *Ccr2* (14), and *Ccr5* (12), and complement *C3* and the complement receptor *Cr2* (15). There is, however, no clear understanding of how these genes, and the pathways they function in, are interconnected, and of the most important drivers of myocardial inflammation. Neither is there a clear understanding of the molecular mechanism of action of immunosuppressive agents in clinical inflammatory cardiomyopathy.

We hypothesized that identifying the gene network activated during the course of murine experimental autoimmune myocarditis, a model of inflammatory cardiomyopathy, would provide a molecular rationale for informing new therapeutic strategies. In this study, we used sequential transcriptome profiling to identify the genetic network underlying inflammatory cardiomyopathy in a mouse model. We identified critical nodes that provide a molecular rationale for current immunosuppressive therapy, and also identified opportunities for future therapy including drugs that could be re-purposed for the treatment of inflammatory cardiomyopathy.

## Materials and Methods

### Mice

BALB/cAnNCrl mice (male, 6–8 weeks old), were purchased from Charles River UK and acclimatized for a week. Male mice were studied as they have a higher severity of myocarditis, reducing animal numbers needed for study (16). Animals were group housed in humidity and temperature-controlled rooms on a 12-h light-dark cycle, and provided food and water *ad libitum*. Mice (8–10 weeks) were immunized as described (17, 18). Peptide Ac-RSLKLMATLFSTYASADR-OH (19) (MyHC-α_614−629_) was obtained at 95% purity from China Peptides. The peptide (1 mg) was dissolved in PBS (1 ml, containing 2%DMSO). Supplemented Complete Freund's Adjuvant (SCFA) was produced by adding 100 mg of powdered heat-killed M. tuberculosis strain H37Ra (MT) (BD, Cat # 231141-6X100 mg) to 25 mL Complete Freund's Adjuvant (Sigma Cat# F5881). SCFA (990 μL) and peptide (1,010 μL) were emulsified using 2.5 mL glass syringes. Control emulsions were prepared by emulsifying SCFA with PBS. Mice were immunized on day 0 and day 7 by injection of 200 μL SCFA + peptide/PBS emulsion (100 μg peptide). Control mice were injected on day 0 and day 7 with 200 μL SCFA + PBS emulsion. Injections of SCFA were performed under isoflurane inhalation anesthesia. All mice also received pertussis toxin (Sigma Cat# P7208, 500 ng/mouse, dissolved in 100 μL PBS) intraperitoneally on day 0. Mice were culled on day 10, 15 and 21 by anesthesia (4% isoflurane inhalation administered in a chamber) followed by cervical dislocation, and hearts were harvested. A portion of the heart was snap frozen in RNA*later* (Ambion, Cat#AM7021) in liquid nitrogen and stored at −80°C for subsequent RNA extraction.

### Cardiac Troponin Measurement

Serum levels of cardiac troponin-I were measured using an iSTAT1 (Abaxis) handheld analyzer and cardiac troponin cartridges (Abaxis) following the manufacturer's instructions.

### Flow Cytometry

Cardiac tissue was digested with collagenase I, XI and hyaluronidase at 37°C for 1 h, and triturated through a nylon mesh to isolate a single cell population as described (14). Red blood cell lysis was performed by adding 10 mL of 1 × red blood cell lysis solution (ACK buffer) with 2 mM EDTA and incubated at room temperature for 3 min. Cells washed with HBSS supplemented with 0.2%(w/v) BSA and 1%(w/v) FCS, centrifuged at 4°C for 10 min at 300 g. Cells were then incubated in blocking solution containing 5% normal mouse serum, 5% normal rat serum, and 1% FcBlock (Cat#553142, BD Biosciences), in PBS on ice for 30 min. Cells were stained with 1:100 dilution of PE anti-mouse CD45 antibody clone 30-F11 PE conjugated (Biolegend, Cat#103106) for 30 min on ice. Cells were washed as described above and analyzed on an Attune Nxt Flow Cytometer with software version 3.1.2. Flow cytometry data were analyzed using FlowJo v10.7.1. The gating strategy used is described in Supplementary Figure 2.

### RNA Extraction

Myocardial RNA was isolated from snap frozen myocardial samples using RNeasy Mini Kit (Qiagen, Cat#74104) following the manufacturer's instructions. Myocardial samples for RNA extraction were chosen to as far as possible satisfy the diagnostic criterion of myocarditis (presence of inflammation) as this may not be 100% penetrant in the BALB/c mouse experimental autoimmune myocarditis (EAM) model (20). Samples used are indicated in Supplementary Figure 1. Total RNA quantity and integrity were assessed using Quant-IT RiboGreen RNA Assay Kit (Invitrogen, Carlsbad, CA, USA) and Agilent Tapestation 2200 RNA Screentape. Purification of mRNA, generation of double stranded cDNA and library construction were performed using NEBNext Ultra II Directional RNA Library Prep Kit for Illumina (New England Biolabs) with minor modifications to manufacturer specifications. Amplified libraries were analyzed for size distribution using the Agilent Tapestation 2200 D1000. Libraries were quantified using Picogreen and relative volumes were pooled accordingly. Sequencing was performed as 75 bp paired end reads on a HiSeq4000 according to Illumina specifications.

### RNA-seq Data Processing and Analysis

Samples were mapped to the GRCm38 genome using HISAT2 (https://ccb.jhu.edu/software/hisat2/index.shtml). Duplicate reads were identified and removed using Picard (https://broadinstitute.github.io/picard/). The mapped reads were then assigned to Ensembl genomic features defined in GRCm38.69 (http://primerseq.sourceforge.net/gtf.html) using feature Counts (http://subread.sourceforge.net/). Analysis was performed using edgeR (version 3.28.1) (https://www.bioconductor.org/packages/release/bioc/html/edgeR.html) and limma (version 3.42.2) packages for R (version 3.6.1). Genes with low read counts across all samples were removed. “Low” read counts were defined as not meeting the equivalent of 10 reads before normalization. If the number of samples where that threshold was met was at least as large as the smallest experimental group (4), then that gene was kept; otherwise the gene was excluded. For each timepoint (Day 10, Day 15, Day 21), the disease and control groups were compared to identify differentially expressed genes. The Benjamini–Hochberg correction was applied to the raw *p*-values to control the false discovery rate (FDR) in the final results.

### Quality Check of RNA Sequences

The Ensembl annotations used for read assignment contained 41,388 genes. We observed at least one read assigned to 27,429 of them across the entire set of data. Under the rules for excluding marginally-expressed genes described above, the number of genes that remain varies by the comparison considered. At 10 days, 14,737 remain, at 15 days, 15,318 and at 21 days, 15,300. The assignment of reads to features for all the (non-outlier) samples are shown in Supplementary Figure 7. Different filters for different time point comparisons—genes with zero count, genes with counts too low in too many samples, and finally the genes taken forward for the analysis are presented in Supplementary Figure 7B.

### Differential Expression, Gene Groupings and Pathway Analysis

Differentially expressed genes between myocarditis and control mice at each time point were defined based on FDR < 0.01 and at least two-fold change in expression level (either induced or repressed). Genes differentially expressed at each time point were compared to each other identifying seven major gene groups (see results), visualized using the UpSetR package (21). Gene groups were subjected to pathway enrichment analysis using the XGR package (22). Pathways and member genes (mouse) were obtained from KEGG (accessed on June 2020). The one-sided Fisher's exact test was used to test the enrichment of pathways for each of gene groups. We carried out such pathway enrichment analysis separately for KEGG Organismal Systems pathways and Environmental Information Processing pathways.

### Network Analysis for Differential Genes Over Time

**Figure 1:**
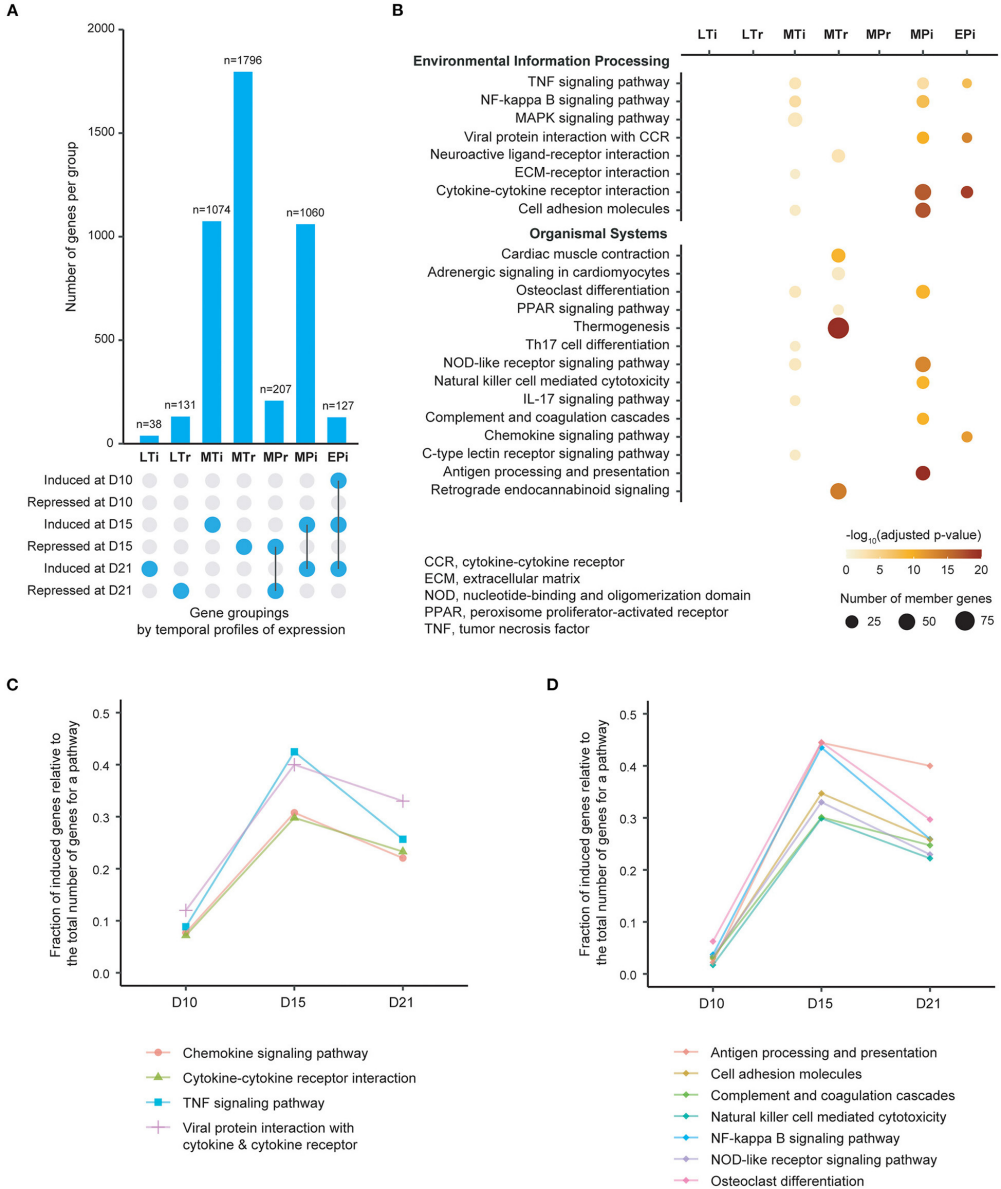
Patterns of gene expression and enrichment of KEGG pathways in the heart over time following induction of experimental autoimmune myocarditis. **(A)** Bar plot showing the number of genes in each of seven gene groups that are classified by onset and persistence of expression. EPi, early-persistent induced; MPi, mid-persistent induced; MPr, mid-persistent repressed; MTi mid-transient induced; MTr, mid-transient repressed; LTi, late-transient induced; LTr, late-transient repressed. D, days from induction of EAM. **(B)** Molecular pathways significantly enriched for each of gene groups based on adjusted *p* < 0.05 (color-coded). The size of each individual dot is proportional to the number of member genes. Pathways are sourced from KEGG maps, and are displayed separately for Environmental Information Processing pathways (top) and Organismal Systems pathways (bottom). **(C)**, Plot of fraction of induced genes (*Y*-axis) within the 4 pathways identified from the EPi group of genes over time (*X*-axis). **(D)** Plot of fraction of induced genes (*Y*-axis) within the 7 pathways identified from the MPi group of genes over time (*X*-axis).

We extended our previous algorithm (heuristically solving a prize-collecting Steiner tree problem) (23) to identify a subnetwork through integrative analysis of time-course expression data and KEGG-merged network interaction data. The algorithm takes two inputs. The first is the score for genes quantifying the expression importance over time, this is, –log_10_(FDR aggregated over 3 time points). The aggregation is based on Fisher's combined method implemented in the dnet package (23); in other words, a gene receives a higher score if it is more significantly changed over time. The second input is the gene interaction network (the parent network) merged from 11 KEGG pathways (illustrated in Figures 1C,D). The output is a gene subnetwork containing highly scored genes that are linked together through a few lowly scored genes as linkers. The subnetwork is highlighted in the parent network for which the layout is rendered using stress majorization (24).

### Attackness and Combinatorial Attack

We used “attackness” to quantify the tolerance of the network to individual node removal, defined as the fraction of network nodes disconnected from the giant component after node removal. The attackness ranges from 0 to 1, with the higher value indicating the more vulnerable (critical) node for the network. Similarly, we introduced a new concept “combinatorial attack” to maximize the attackness for nodes in a specific combination removed, for example, identifying the optimal combination involving any two nodes.

### Drug Repurposing Based on ChEMBL

We extracted current drug therapeutics, target genes and disease indications from the ChEMBL database (25) (v27), considering: (1) non-withdrawn drugs; (2) non-promiscuous therapeutic target genes with well-defined mechanism of action and explaining the efficacy of drugs in disease; and (3) drugs with the maximum phase for a gene given a disease indication.

### Statistical Analysis

Summary statistics were calculated in GraphPadPrism, and statistical significance of differences between groups were evaluated using a one-way ANOVA with Dunnett's correction for multiple comparisons or using an unpaired *t*-test as indicated. The Benjamini–Hochberg correction was applied to the raw *p*-values to control the false discovery rate (FDR) in the RNASeq results.

### Ethics Declarations

All animal procedures were approved locally by the University of Oxford Animal Welfare and Ethical Review Body and by the UK Home Office and carried out in accordance with the UK Animals (Scientific Procedures) Act 1986, under project license PPL P973A60F5. All procedures conformed to guidelines from Directive 2010/63/EU of the European Parliament on the protection of animals used for scientific purposes.

### Data Availability

The datasets supporting the conclusions of this article are available in the Gene Expression Omnibus repository, with accession number GSE155423. Codes and associated data are provided as Supplementary Material.

## Results

### Experimental Autoimmune Myocarditis

We sought to determine how the cardiac transcriptome changes during autoimmune myocardial inflammation using an established model of experimental autoimmune myocarditis (EAM) in which heart-specific antigens are used to trigger myocarditis (3, 18). We induced EAM in mice by immunizing them with myosin heavy chain peptide and adjuvants (Complete Freund's Adjuvant and pertussis toxin, see schematic in Supplementary Figure 1A and methods). Control mice were generated by substituting PBS vehicle for myosin heavy chain peptide. We performed flow cytometry analysis (FACS) of single cell suspensions from hearts isolated at day 21 following immunization to determine the proportion of CD45^+^ (total leucocyte) cells as a quantitative measure of inflammation, in these mice, and also in naïve mice. We found that there was a significant increase in myocardial CD45^+^ cells in EAM mouse hearts (Supplementary Figures 1B, 2). All EAM hearts had elevated levels of CD45^+^ cells exceeding the 95% confidence interval of either the control or the naïve group, and 4 of 6 EAM mice were chosen for RNA sequencing. There was no significant difference in CD45^+^ cells between naïve and control mice (Supplementary Figure 1B). We next analyzed EAM mice at day 15 and day 10, again by FACS of single cell suspensions from hearts (Supplementary Figures 1C,D), and also by measuring cardiac troponin I (cTnI) (Supplementary Figures 1E,F). There was a statistically significant difference in myocardial CD45^+^ cells at day 15 between EAM and control mice (Figure 1C), and also a significant elevation of cTnI, indicative of myocardial damage (Supplementary Figure 1E). None of the EAM mice at day 10 showed elevated levels of either myocardial CD45% cells or cardiac troponin I (Supplementary Figures 1D,F). At each time point 4 mice in EAM and control groups were chosen for RNA sequencing.

### RNA Sequencing Shows That Major Inflammatory Pathways Are Activated Early in the Course of Autoimmune Myocardial Inflammation

We identified genes that were differentially expressed between immunized and control groups at each time point using a false discovery rate (FDR) threshold of <0.01 and at least two-fold changes (either induced or repressed). Based on their temporal profiles and degree of persistence of expression over time, we then categorized these genes into seven major groups (Figure 1A, Supplementary Figure 3). For each gene group we next performed enrichment analysis using well-curated pathways based on the KEGG map (26) (Figure 1B). Twenty-two significantly enriched pathways (adjusted *p* < 0.05) were identified, with 11 pathways being identified from groups with persistent gene induction (Supplementary Figures 4, 5). Of these 11 pathways, four (cytokine-cytokine receptor, viral protein interaction with cytokine–cytokine receptor, TNF and chemokine signaling pathways) are activated early in the disease process (day 10) at a time point where there is no evidence of leucocyte infiltration (Figure 1C). There is substantial further activation of these four pathways at day 15 followed by a decline in activity by day 21, despite increased numbers of infiltrating leukocytes. Seven pathways are predominantly activated at day 15 (Figure 1D). These include antigen processing and presentation, cell adhesion molecules, complement and coagulation, NK cell mediated cytotoxicity, NF-kB signaling, NOD-like signaling and osteoclast differentiation. These results indicate that inflammatory pathways are activated early in the course of autoimmune myocardial inflammation, and molecular changes may precede the onset of leucocyte infiltration.

### Integration of Gene Expression Changes With KEGG Molecular Interactions Identifies a 50-Gene Network in Autoimmune Myocardial Inflammation

**Figure 2:**
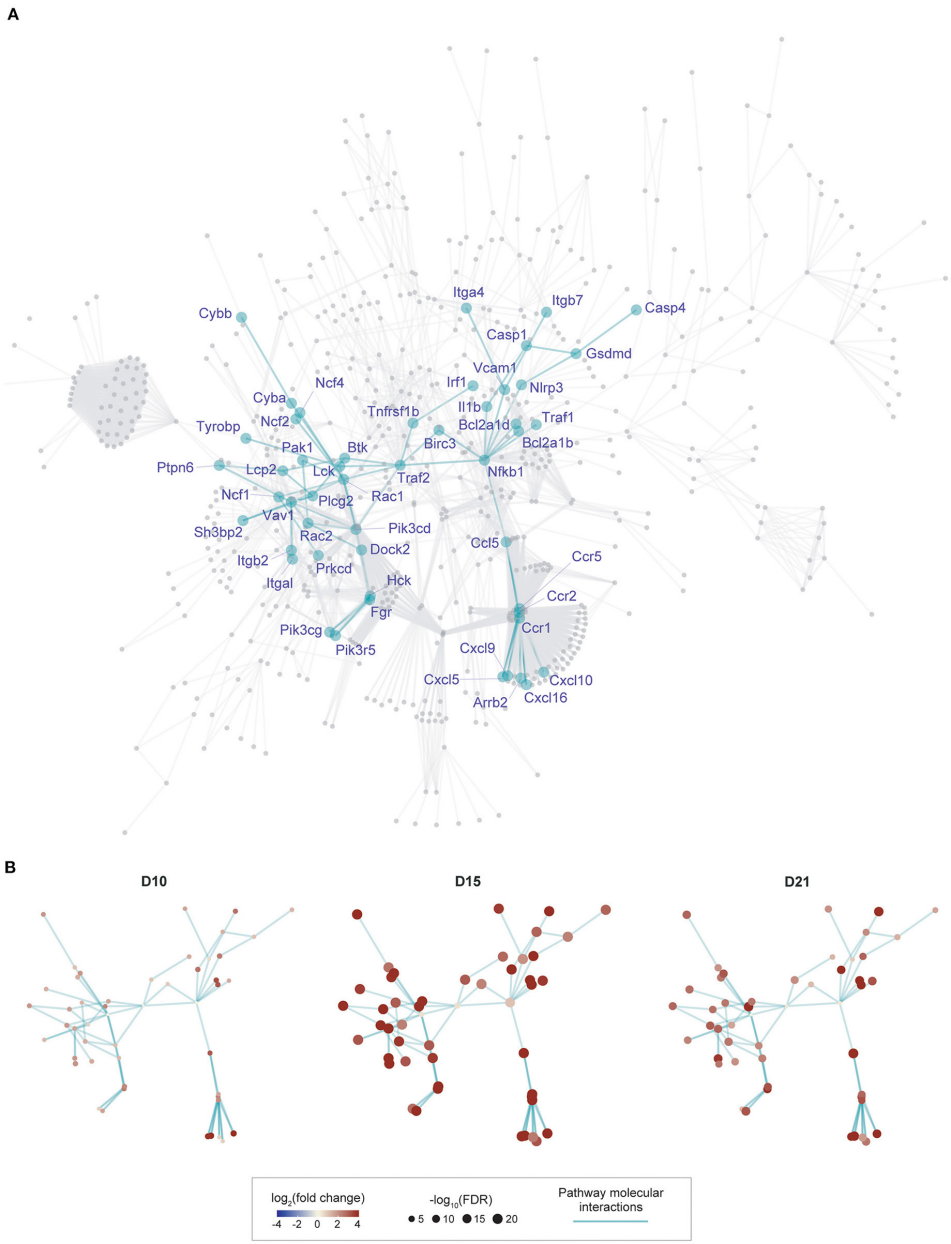
Gene subnetwork identification and temporal evolution in murine myocarditis model. **(A)**, Overview of the gene subnetwork, with 50 gene nodes labeled by gene symbols and 75 connections between nodes (network edges). This subnetwork was identified from a parent network (in gray, obtained by merging 11 KEGG pathways identified in Figure 1) by scoring genes for their expression importance over time in EAM. Genes known to be required for the development of murine myocarditis are indicated in boxes. **(B)**, Gene subnetwork illustrated at three different time-points with the same layout as shown in **(A)**. Nodes at each time point are colored by log_2_(fold change) and sized by –log_10_(FDR) as indicated in the scale.

Although the 11 pathways identified above are informative individually, it is unclear how genes from these pathways are networked as a whole, and which part of the network formed by these pathways is best reflective of our context-specific data (i.e., time-course expression data generated in this study). We reasoned that exploring the topology of this network in an unbiased manner could better understand disease mechanism. We therefore performed an integrated analysis of KEGG molecular interactions with gene expression changes at different time points (see online methods), identifying a subnetwork of 50 gene nodes based on their expression importance over time (Figure 2A). The cascade of subnetwork activation through the course of the disease process in this model of myocarditis is visualized in Figure 2B, where fold change over time and differential significance of individual genes are indicated respectively by color and size of individual gene nodes. Notably, interacting genes in this subnetwork are all induced with the changes peaking at the intermediate time point (day 15).

### Modest Network Disconnection Is Achieved by Single Node Removal

**Figure 3:**
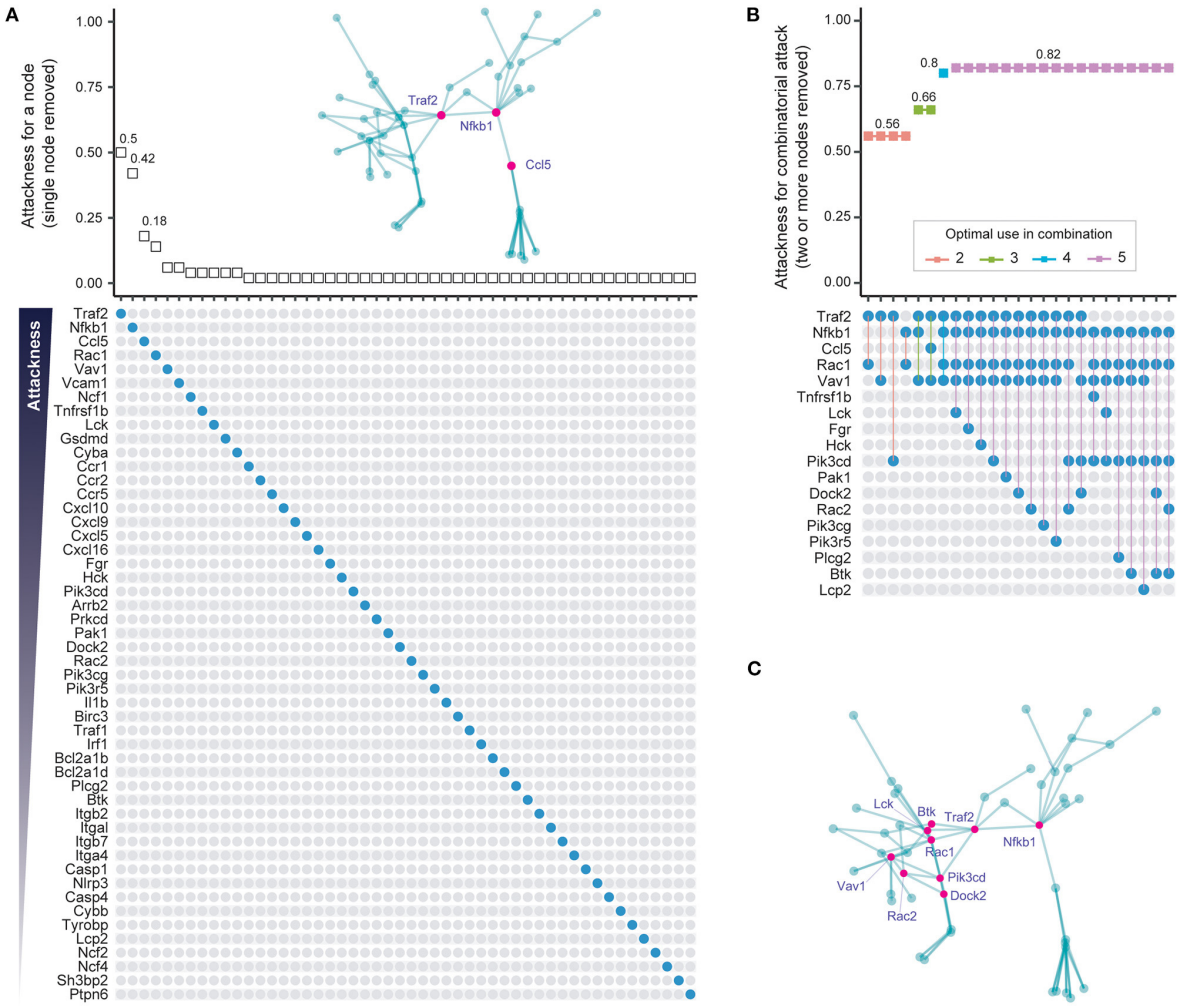
Individual and combinatorial attack to identify vulnerable subnetwork nodes. **(A)** Individual attack strategy. *Top panel*: Plot of attackness (*Y*-axis) by node (*X*-axis, nodes ordered as in bottom panel). The “attackness” metric quantifies the susceptibility of the subnetwork to the individual node removed, and is defined as the fraction of nodes disconnected from the giant component after node removal. *Bottom panel*: Nodes in the 50-node subnetwork are illustrated as colored circles in descending order of attackness. *Inserted panel (top)*: Graph of the 50-node subnetwork with the three nodes with highest attackness values indicated in pink. **(B)** Combinatorial attack strategy to identify node combinations that maximize network disconnection. *Top panel*: Plot of combinatorial attackness (*Y*-axis) by node combinations (*X*-axis, node combinations indicated in bottom panel). *Bottom panel*: Nodes removed in combination are indicated by connected colored circles. **(C)** Graph of the 50-node subnetwork with the nine nodes used most frequently in combinatorial attack indicated as pink circles.

One way of identifying the importance of a node is to quantify the tolerance of the network to node removal based on well-established percolation theory [reviewed in (27)]. A measure of overall network connectivity, and hence function, is the size of the largest connected, or “giant” component (28). The effect of node removal can be defined in terms of the fraction of network nodes disconnected from the giant component after node removal, a measure that we call “attackness” for simplicity. According to the percolation process, removal of a node with high attackness results in a large proportion of disconnected nodes. To assess the potential utility of this approach, we analyzed the 50-gene subnetwork to calculate attackness for each node (Figure 3A). We find that *Traf2* and *Nfkb1* have the highest degree of “attackness” (0.5 and 0.42, respectively).

### Maximal Network Disconnection Is Achieved by Removal of *Traf2, Nfkb1, Rac1*, and *Vav1*

Most diseases, however, typically require a polypharmacological approach, where multiple targets are attacked (29). To minimize on-target adverse effects it is important to identify the smallest possible number of nodes that need to be targeted in order to have an adequate effect. To address this, we removed between two to five nodes in combination and assessed the effect of such removal on the fraction of network nodes disconnected from the giant component (i.e., the combinatorial attackness). The top-ranking combinations and their corresponding combinatorial attackness are shown in Figure 3B, with genes frequently involved in these top combinations highlighted in the subnetwork (Figure 3C). Removal of four nodes (*Traf2, Nfkb1, Rac1*, and *Vav1*) is predicted to disconnect 80% of nodes from the giant component. Combined attack on *Nfkb1* and *Rac1* is predicted to disconnect 56% nodes in the 50-node network.

### Combinatorial Attack Identifies Additional Nodes Targeted by Approved Therapeutics

**Figure 4:**
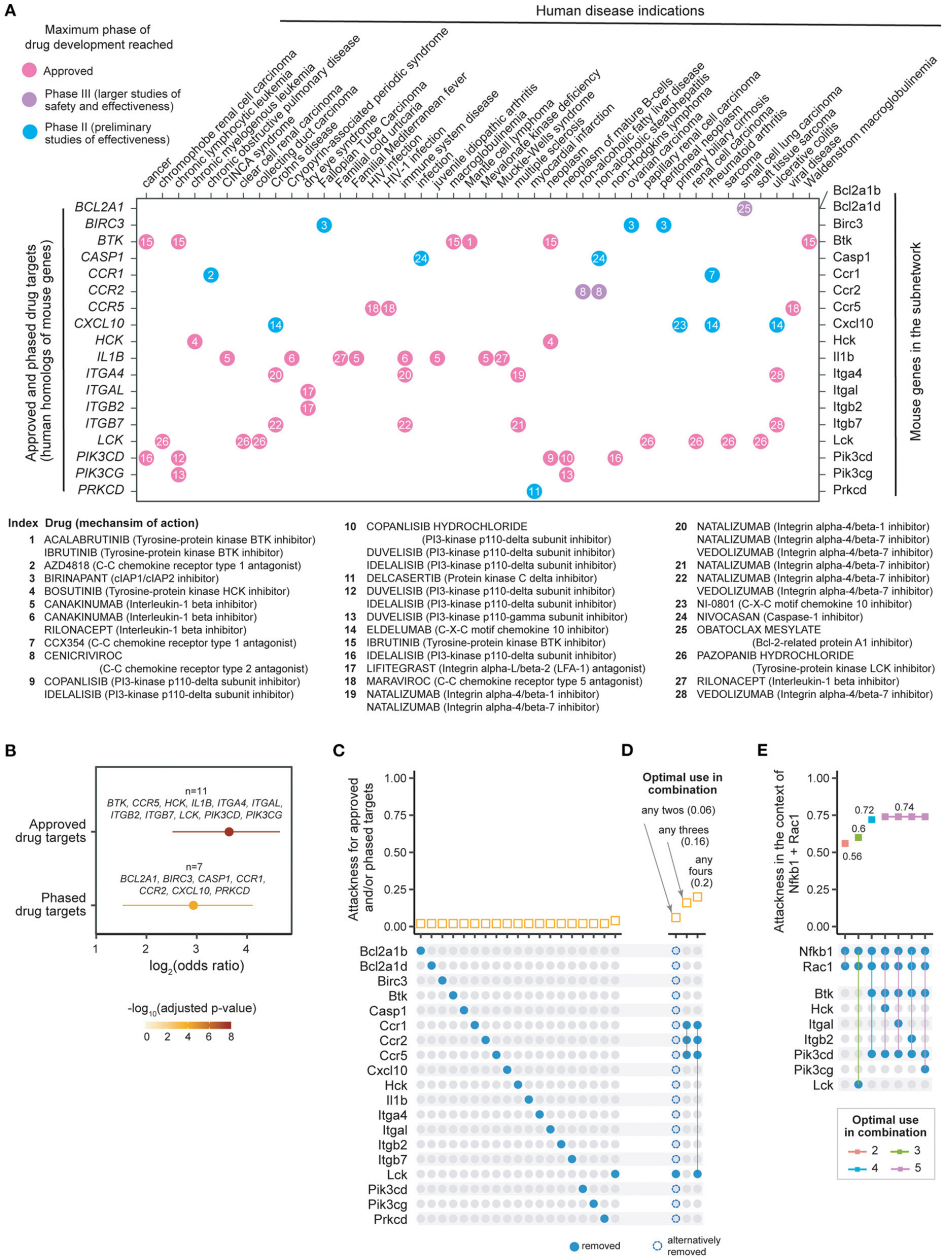
Individual and combinatorial attack strategies to identify re-purposing candidates. **(A)** Dot plot showing 19 mouse genes in the subnetwork (*X*-axis, right) and their human homologs (*Y*-axis, left) mapped to currently approved and phase II/III drugs in human disease indications (indicated on the *X*-axis, and obtained from ChEMBL v25). Dots are color-coded by clinical phase status as indicated in the legend. Information on drugs and mechanism of action is indexed in the plot and detailed beneath the dot-plot. **(B)** Forest plot of approved or phased drug targets enriched in the subnetwork genes. The significance level (adjusted *p*-value), odds ratio and 95% confidence interval (represented by lines) calculated according to Fisher's exact test (one-sided). Also illustrated are gene lists overlapped. **(C)** Individual attack strategy. *Top panel*: Plot of attackness (*Y*-axis) by node (*X*-axis, nodes indicated by colored circles in the bottom panel). *Bottom panel*: 19 nodes within the 50-node subnetwork that have identifiable drugs. **(D)** Combinatorial attack strategy to identify node combinations that maximize network disconnection. *Top panel*: Plot of combinatorial attackness (*Y*-axis) by node combinations (*X*-axis, node combinations indicated in bottom panel). *Bottom panel*: Nodes removed in combination are indicated by connected colored circles. **(E)** Combinatorial attack strategy to identify additional node combinations that maximize network disconnection in the context of *Nfkb1* and *Rac1* node removal using state-of-the-art therapy (glucocorticoid and azathioprine). *Top panel*: Plot of combinatorial attackness (*Y*-axis) by node combinations (*X*-axis, node combinations indicated in bottom panel). *Bottom panel*: Nodes removed in combination are indicated by connected colored circles.

Drug repurposing is a strategy that repositions existing effective therapeutics with acceptable safety profiles to new indications (30). We used the ChEMBL database (25) to explore the evidence supporting potential therapeutics targeting products of the human gene equivalents of the mouse subnetwork. We identified 11 genes (*BTK, CCR5, HCK, IL1B, ITGA4, ITGAL, ITGB2, ITGB7, LCK, PIK3CD*, and *PIK3CG)* that could be targeted by drugs already in clinical use (Figure 4A). We also identified 7 preclinical (phased) drug targets. These include two genes (*BCL2A1* and *CCR2*) targeted by drugs in clinical phase III, and five genes (*BIRC3, CASP1, CCR1, CXCL10*, and *PRKCD*) targeted by drugs in clinical phase II. We found the highest degree of support from clinical evidence (approved therapeutics), and also observed enrichment for preclinical (phased) drug targets, though at a less significant level (Figure 4B). We next calculated the attackness for these targets and found the lowest degree for all of them except for *Lck* (Figure 4C). This observation motivated us to explore the effect of a combinatorial attack strategy on the subnetwork. The top two-node, three-node and four-node combinations and their corresponding combinatorial attackness are shown in Figure 4D. We find that the tyrosine-protein kinase *LCK* inhibitor can be combined with any other inhibitor to achieve a small improvement in effect. A further improvement in effect may be observed by combining C–C chemokine receptor type 1 (CCR1) antagonist (Phase II), C–C chemokine receptor type 2 (CCR2) antagonist (Phase III), and C–C chemokine receptor type 5 (CCR5) antagonist (approved). The effect of this triplet combination can be further enhanced with addition of the LCK inhibitor.

### Combinatorial Attack in the Context of Established Therapy Identifies *Btk* and *Pik3cd*

As discussed above, established therapy with prednisolone and azathioprine, targeting *Nfkb1* and *Rac1*, respectively, is predicted to disconnect 56% nodes in the 50-node network. We evaluated a repurposing strategy in the context of *Nfkb1*+*Rac1* node removal. We find that the targeting *Btk* and *Pik3cd* in the context of established therapy will disconnect 72% of the nodes in the 50-node network, a substantial increase from which there is no further improvement by targeting another node (Figure 4E).

## Discussion

The RNA sequencing studies reported here provide a resource with demonstrated utility in advancing our knowledge of the molecular pathology of autoimmune myocardial inflammation. Our work advances published studies of molecular pathology by integrating the temporal sequence of gene expression changes with well-established information on how these genes interact in pathways. Supporting our analysis, several genes within the 11 pathways identified in this study have already been shown to be critical for the development of EAM, including *Ccl2* (12), *Ccl3* (12), *Ccr2* (14), *Ccr5* (12), *Il1r1* (9), *Il17ra* (31), and *Tnf* (5). The role of NK cells and complement pathways in myocarditis has also previously been elucidated (15, 32). Importantly, a number of genes and pathways are identified that are induced at early time points and persist through the course of inflammation. Our work advances the molecular pathology of autoimmune myocardial inflammation by integrating these genes and pathways into a single network and identifying a 50-gene molecular network that is induced over the time course of autoimmune myocardial inflammation. Elements within this molecular network have potential for exploitation as biomarkers or molecular signatures of disease. The diagnosis of myocarditis and inflammatory cardiomyopathy currently relies on the presence of leucocyte infiltration in endomyocardial biopsy specimens, which is the “gold-standard” (2). As such infiltrates may be patchy, the disease may go unrecognized.

The data from RNA sequencing analysis indicate that chemokine signaling pathway and cytokine–cytokine receptor interactions are elevated at D10, and increase further by D15 and at D21. While no evidence of myocardial inflammation is detected at D10, it is clearly detected at D15 and peaks by D21. Our results thus show that molecular changes in the myocardium appear before leucocyte infiltration occurs, and persist through to later stages. The delay between chemokine/cytokine gene expression and maximum accumulation of CD45^+^ cells is expected, and likely arises from the necessary subsequent stages of chemokine protein translation, post-translational modification, secretion, presentation on the endothelial luminal surface, binding to leucocyte chemokine receptor, activation of the leucocyte adhesion cascade, and leucocyte transendothelial migration that leads to tissue leucocyte accumulation (33, 34).

Alternative approaches that could be used for such molecular phenotyping include RT-qPCR, proteomic approaches such as western blotting or multiplexed immunoassay (e.g., Luminex xMAP) technologies, and single-cell RNA sequencing approaches to characterize the nature of the immune cell infiltrate. RNA sequencing is considered the “gold-standard” for whole transcriptome gene expression studies, and is highly correlated with alternative transcriptomic approaches that detect changes in expression of individual genes such as microarrays or RT-qPCR (35). Proteomic approaches such as western blotting or multiplexed immunoassays we consider impractical for the molecular characterization of multiple gene products due to the very small amounts of tissues that are typically obtained in myocardial biopsy specimens, and the requirement for highly specific and sensitive antibodies to the gene products of interest.

A limitation of our work is that the restricted amount of tissue available for FACS analyses (most tissue being used for RNA extraction), made more detailed studies of cellular subpopulations technically challenging. In future studies characterization of inflammatory subpopulations—for instance using single cell RNA sequencing—would be a desirable goal, as it would allow an understanding of the changes in cellular compartments, and also allow correlation with transcriptomic analyses. The application of single-cell RNA sequencing as a molecular diagnostic tool is indeed of great interest, and will likely provide complementary information to bulk RNA sequencing. Single-cell RNA sequencing is technically more challenging and substantially more expensive than bulk RNA sequencing, and currently may be impractical for diagnostic applications where very small amounts of tissue are typically available for study. Taken together, we suggest that molecular characterization of biopsied myocardium using bulk RNASeq may be a sensitive and cost-effective indicator of autoimmune myocardial inflammation in comparison to histopathology, and could complement histopathology in the evaluation of myocardial inflammation.

These studies also provide a molecular rationale for immunosuppressive therapy of inflammatory cardiomyopathy. Recommended immunosuppressive approaches for autoimmune and virus-negative inflammatory cardiomyopathy employ glucocorticoids, azathioprine and cyclosporine singly or in combination (1, 2). These immunosupressive approaches either directly or indirectly target three of these four critical nodes identified by the combinatorial attack strategy. The azathioprine metabolite 6-thio-GTP directly binds RAC1 to suppress VAV1 guanosine exchange activity on RAC1 and inhibits T-cell-APC conjugation, causing immunosuppression (36, 37). Glucocorticoids such as prednisolone repress NFKB activity through diverse mechanisms including increasing its cytoplasmic retention, and reducing its synthesis (38). Cyclosporine also inhibits NFKB activity through its action on the 20S proteasome (38). Combined attack on NFKB and RAC1 is predicted to disconnect 56% nodes in the 50-node network. These findings support the validity of the subnetwork definition coupled with node disconnection approach developed here to predict therapeutic targets, and suggests that the extent of network disconnection achieved predicts therapeutic response. These findings thus provide a molecular rationale for the immunosuppressive therapy recommended currently for inflammatory cardiomyopathy.

While the combinatorial attack strategy predicts critical network node/target combinations for drug therapy, it does not necessarily imply therapeutic success. Such a strategy may even result in the use of drugs with unacceptable on-target adverse effects. For instance, while TRAF2 has a key role in the subnetwork, its loss results in inflammatory and cardiac failure phenotypes which would invalidate it as a target (39, 40). Drug repurposing is a strategy that repositions existing effective therapeutics with acceptable safety profiles to new indications (30). As discussed above, established therapy with prednisolone and azathioprine, targeting *Nfkb1* and *Rac1*, respectively, is predicted to disconnect 56% nodes in the 50-node network. Given that any new therapeutic can only be ethically tested in the context of combination with established therapy, we evaluated a repurposing strategy in the context of *Nfkb1*+*Rac1* node removal. We find that the targeting *Btk* and *Pik3cd* in the context of established therapy will disconnect 72% of the nodes in the 50-node network, which predicts a substantial improvement in therapeutic efficacy. These *in silico* predictions need to be validated in appropriate studies, e.g., using genetic (e.g., knockout) or pharmacological (e.g., using agents such as Ibrutinib and Idelalisib, targeting BTK and PIK3CD, respectively) approaches in animal models of inflammatory cardiomyopathy.

In summary we have conducted a series of analyses (Supplementary Figure 6), starting with RNA sequencing to define the sequential evolution of the myocardial transcriptome during experimental autoimmune myocarditis. Combining the data with KEGG pathways allows identification of a myocarditis gene subnetwork that is induced at early, mid and late time points through the course of cardiac inflammation. We show that key vulnerable nodes in the temporally evolving subnetwork can be identified by a combinatorial attack strategy, the concept being based on well-established percolation theory. This approach is supported by the identification of the key nodes *Nfkb1* and *Rac1*, in the myocardial inflammation subnetwork, which are well-established targets of glucocorticoids, cyclosporine and azathioprine, and which have proven efficacy in the therapy of autoimmune and virus-negative inflammatory cardiomyopathy. Our analysis provides an *in silico* prediction that agents such as Ibrutinib and Idelalisib, targeting BTK and PIK3CD, respectively, could be re-purposed for the therapy of virus-negative inflammatory cardiomyopathy. Combination therapy is increasingly adopted across a range of chronic immuno-inflammatory diseases with the aim of inducing sustained remission (41, 42). Our approach, combining sequential transcriptome profiling, in-depth pathway network analysis and combinatorial attack, adds a new strategy to the repository of network medicine analyses designed to aid development of drug repurposing and drug combinations (43, 44). We suggest that our approach would be applicable more broadly to other immuno-inflammatory disorders where a similar series of analyses could be applied not only in mouse models, but also in human disease.

## Data Availability Statement

The datasets supporting the conclusions of this article are available in the Gene Expression Omnibus repository, with accession number GSE155423. Codes and associated data are provided as a series of online chapters at the project web interface (http://galahad.well.ox.ac.uk/Myocarditis).

## Ethics Statement

All animal procedures were approved locally by the University of Oxford Animal Welfare and Ethical Review Body and by the UK Home Office and carried out in accordance with the UK Animals (Scientific Procedures) Act 1986, under project license PPL P973A60F5. All procedures conformed to guidelines from Directive 2010/63/EU of the European Parliament on the protection of animals used for scientific purposes.

## Author Contributions

KS and GD performed and analyzed mouse and flow cytometry experiments. HF, JK, and SB conceived the network analysis strategy. HF designed all scripts for network analysis. BW and HL designed the RNA sequencing study and analyzed RNA sequencing data. SB, DC, RW, and JK conceived the study and obtained funding support. KS, HF, JK, and SB drafted the manuscript. We thank Dr. Ying-Jie Wang for his advice on FACS data analysis. All authors contributed to editing the manuscript and approved the final manuscript version.

## Conflict of Interest

The authors declare that the research was conducted in the absence of any commercial or financial relationships that could be construed as a potential conflict of interest.
