# Supplementary material for: Transcriptomic Analysis of Inflammatory Cardiomyopathy Identifies Molecular Signatures of Disease and Informs in silico Prediction of a Network-Based Rationale for Therapy
Source: Front Immunol. 2021 Mar 5;12:640837. doi: 10.3389/fimmu.2021.640837 (PMC7973371; doi:10.3389/fimmu.2021.640837)
Supplement: Supplementary file 1 [file Data_Sheet_1.pdf]

Singh et al. Supplementary Information

Availability of data and materials

The datasets supporting the conclusions of this article are available in the Gene Expression Omnibus repository, with accession number GSE155423.

Codes. Codes and associated data are provided as a zip compressed supplementary file.

Chapter 1: Overview
Chapter 2: RNAseq analysis (quality control and differential analysis)
Chapter 3: List of differentially expressed genes
Chapter 4: Packages required
Chapter 5: Gene groupings (5.1 R function Upset, 5.2 group visualisation, 5.3 grouped genes, 5.4 heatmap visualisation)
Chapter 6: Pathway analysis (6.1 enrichment analysis, 6.2 enriched pathways),
Chapter 7: Subnetwork analysis (7.1 subnetwork identification, 7.2 subnetwork visualisation, 7.3 gene nodes in the subnetwork, 7.4 edges in the subnetwork),
Chapter 8: Combinatorial attack analysis (7.1 R function CombAttack, 7.2 individual node, 7.3 two-node combination examples).
Chapter 9: R session information
Chapter 10: Flow cytometry data

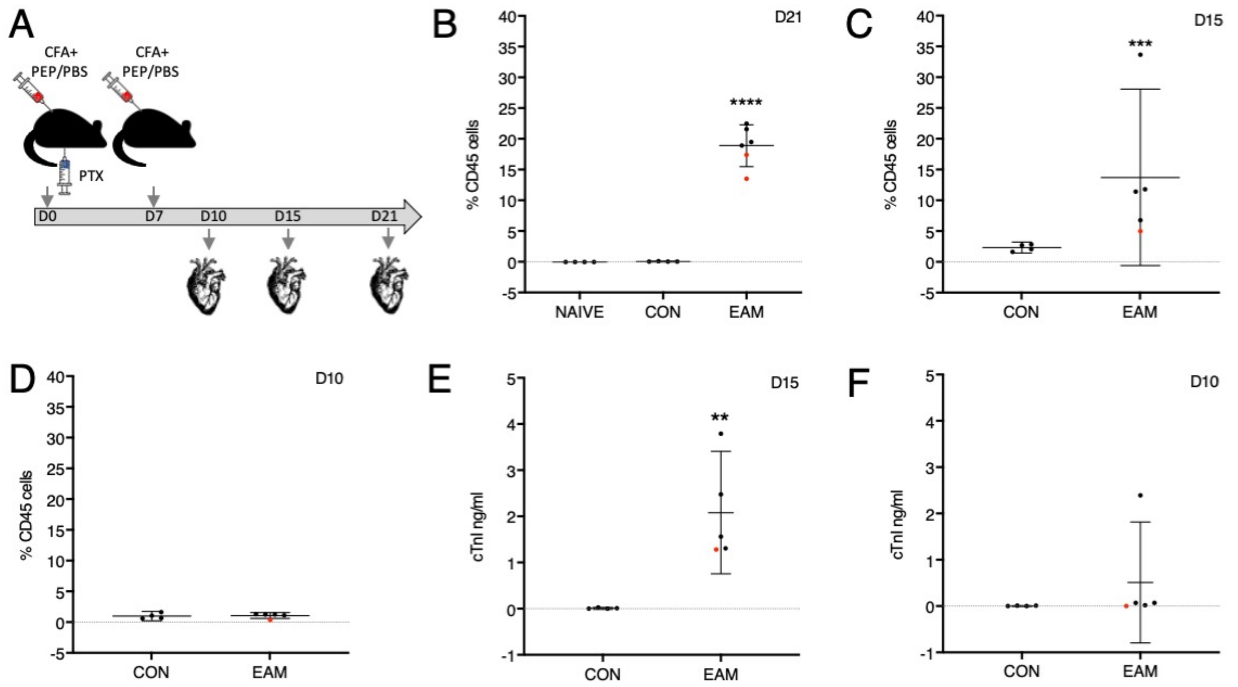

Figure S1 Experimental autoimmune myocarditis

A, Scheme illustrating the generation of mice with experimental autoimmune myocarditis (EAM) and controls (CON). Complete Freund's Adjuvant (CFA) and *MyHCa* peptide (PEP, EAM) or phosphate buffered saline (PBS, CON) were administered subcutaneously. Pertussis toxin (PTX) was administered intraperitoneally. D indicates day of protocol. Hearts were harvested at the indicated time points.

B-D, Summary data for flow cytometry analysis of single cell populations isolated from mouse hearts at the indicated time points. Y axis shows CD45+ (leucocytes) cells as a percentage of the single cell population.

E, F, Summary data for serum Troponin I (cTnI) analysis at the indicated time points. Y axis shows cTnI (ng/ml).

Data in B-F are presented as mean ± 95% confidence interval, and with individual data points indicated. All measurements were taken from distinct samples. Data points in black represent data from mice that were subsequently used for RNA-seq analysis (n= 4 for control mice at each time point, n=4 (of 6) EAM at D15, and n=4 (of 5) EAM at D15 and D10). Statistical significance of differences between EAM and CON groups were evaluated using a one-way ANOVA with Dunnett's correction for multiple comparisons (B) or using an unpaired t-test (D-F), and significance indicated by asterisks: ** $P \leq 0.01$, *** $P \leq 0.001$, **** $P \leq 0.0001$.

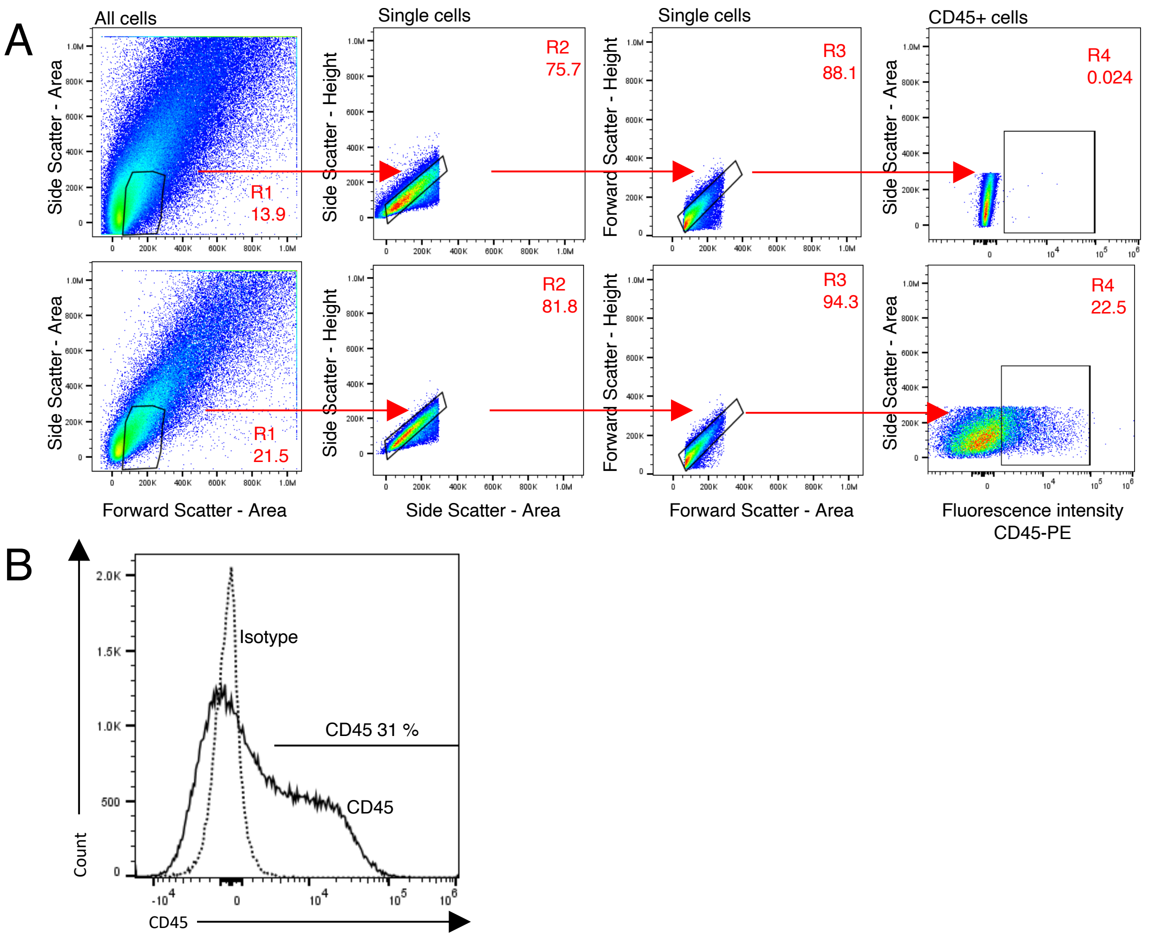

Figure S2. Flow cytometry analysis

A, Representative flow cytometry dot-plots of myocardial cells. *Top panels*: control, *bottom panels*: myocarditis. Panel series from left to right show all cells, single cells and CD45+ cells. Boxed areas in each dataset represent the cell type. Arrows indicate the serial gating strategy used to identify different cell populations. Cell debris was eliminated using forward scatter area (FSC-A) vs side scatter area (SSC-A) (gate R1). Cells in gate R1 were gated for single cells to remove doublets and clumps using Side scatter area (SSC-A) vs Side scatter height SSC-H (gate R2) followed by forward scatter area FSC-A vs forward scatter height FSC-H (gate R3). Cells in R3 were gated for expression of leucocyte marker, using anti-CD45 PE antibody (gate R4). Percentages of cells in each gate are indicated.

B, Representative flow cytometry histogram of CD45 +staining in myocarditis heart. Y-axis shows cell count (side scatter), and x-axis the fluorescence intensity on log scale. Dotted line is the isotype control used to determine background for CD45 antibody. Regular line shows staining for CD45 positive cells. % of cells exceeding fluorescence intensity in comparison to isotype control (i.e. CD45 cells) are indicated.

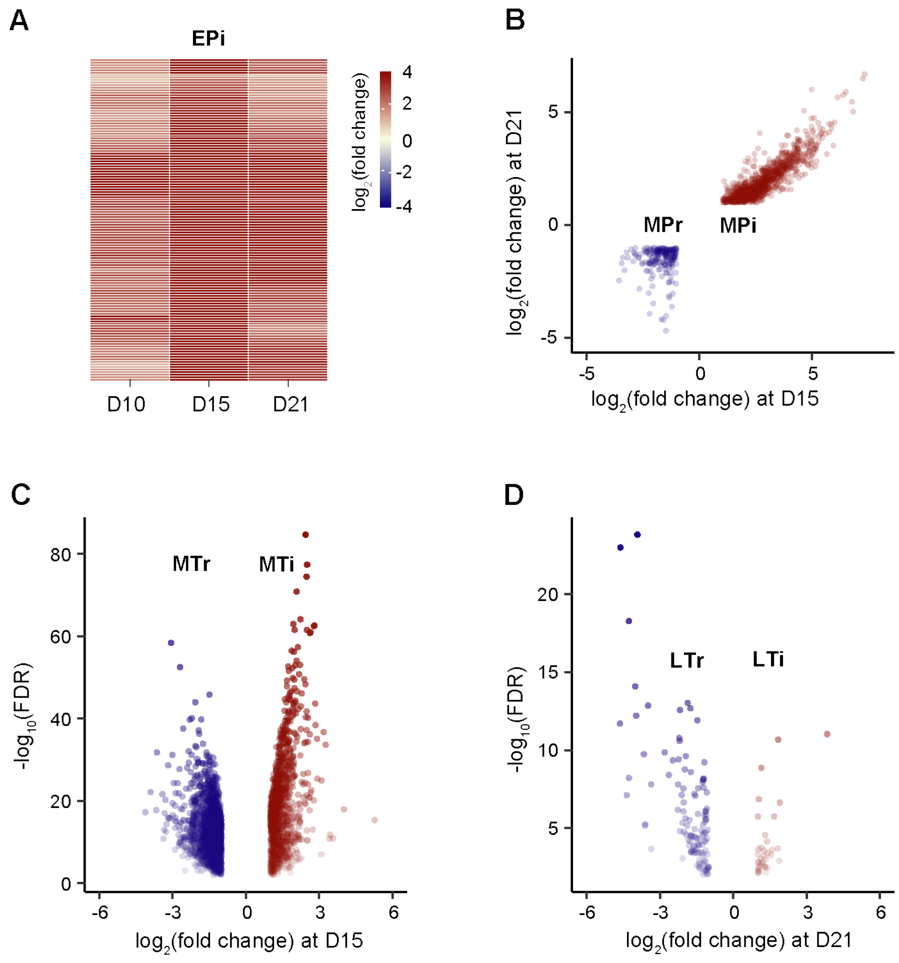

Figure S3 Patterns of gene expression in experimental autoimmune myocarditis.
A, Heatmap illustrating EPi genes with persistent induction at all three time points. **B**, Scatter plot showing MPi and MPr genes at D15 (on the X-axis) and D21 (on the Y-axis). **C, D**, Volcano-like plots illustrating MTi and MTr genes (**C**) and LTi and LTr genes (**D**).

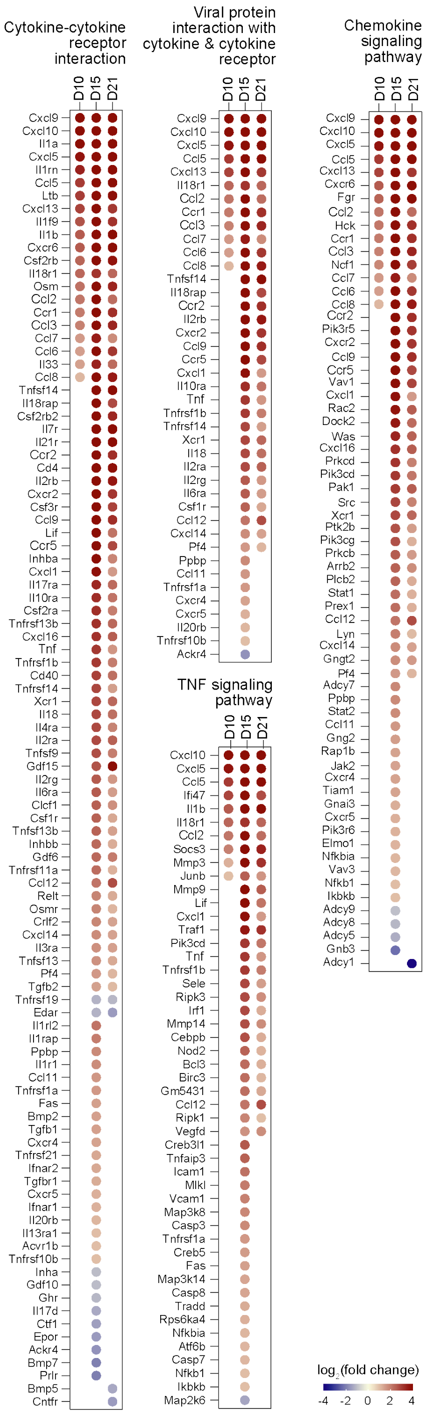

Figure S4 KEGG pathways enriched in group EPi.
Heat maps of individual genes at D10, D15 and D21 within the 4 pathways identified from the EPi group of genes. For a pathway, only significantly expressed genes (FDR <0.01 plus either induced or repressed at least two-fold changes) at the indicated time point are shown, color-coded by log_2(fold change) and ordered by similarity of expression pattern over time.

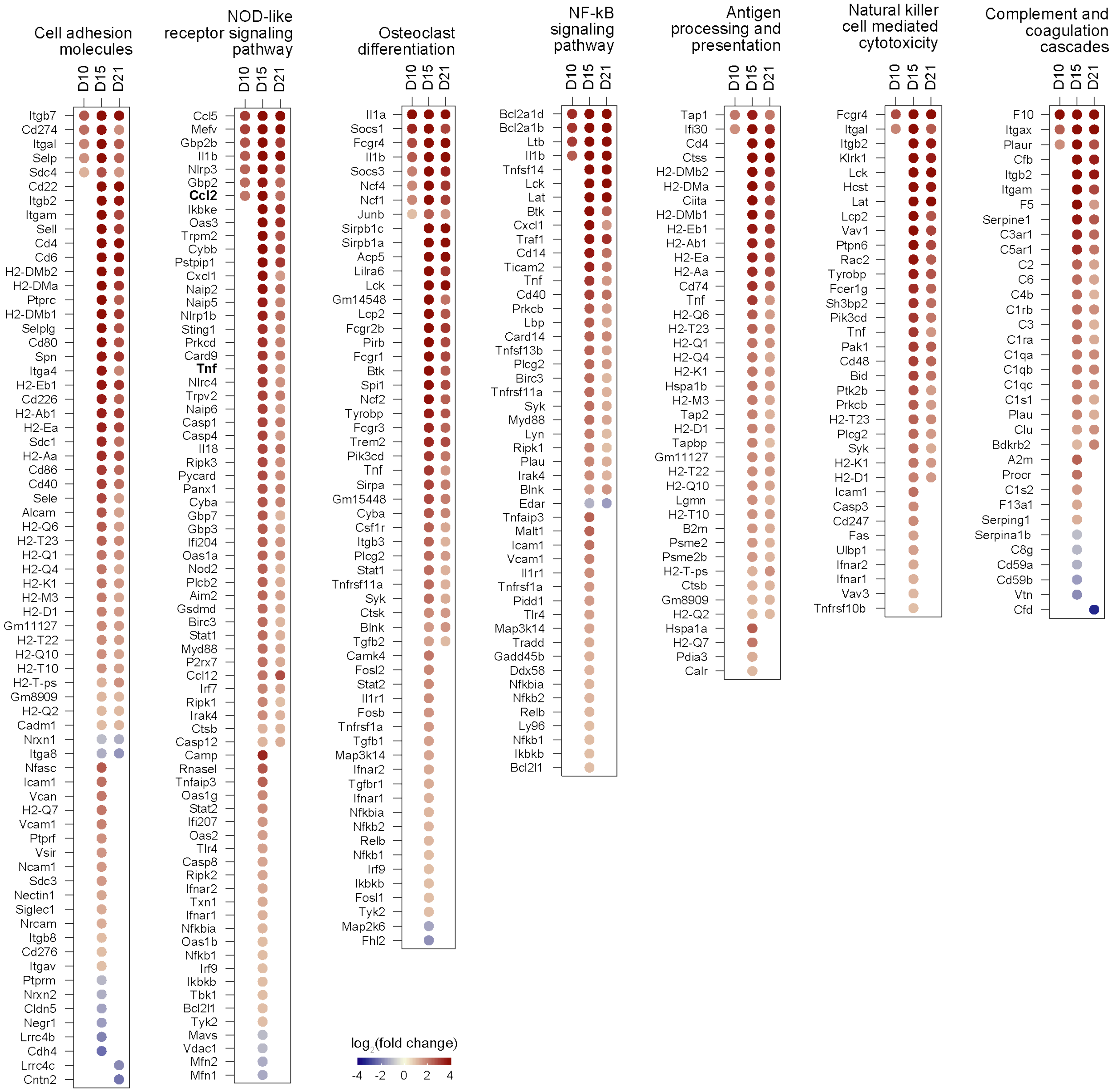

Figure S5 KEGG pathways enriched in group MPi. Heat maps of individual genes at D10, D15 and D21 within the 7 pathways identified from the MPi group of genes. For a pathway, only significantly expressed genes (FDR <0.01 plus either induced or repressed at least two-fold changes) at the indicated time point are shown, color-coded by log_2(fold change) and ordered by similarity of expression pattern over time.

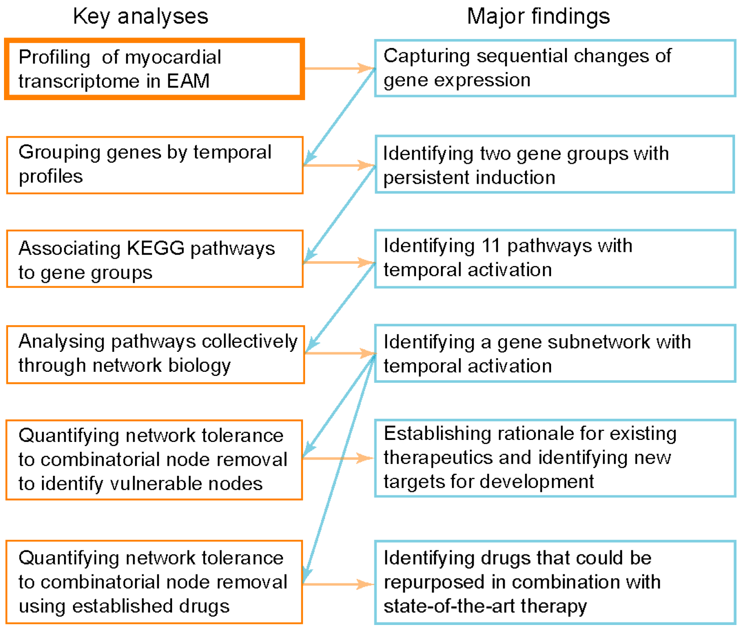

Figure S6. Summary of key analyses and major findings.

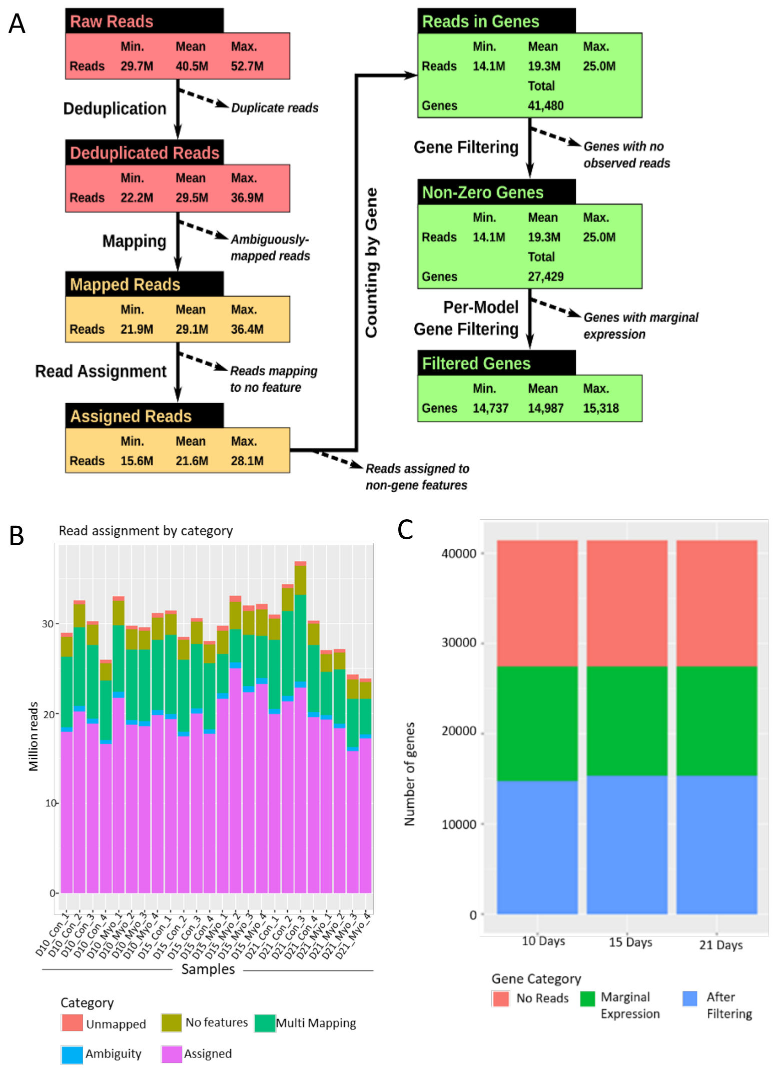

Figure S7 RNA Sequencing Data Analysis and Quality Control

A, *Left panel*: Processing steps from raw reads to assigned reads, showing numbers of reads at each step. Ranges of reads (min, mean and max) are across samples. Reasons for discarding reads are provided at each step. Details of read assignment by category following the mapping step is shown in B.

Right panel: Processing steps from assigned reads to genes used in the analysis, showing numbers of reads and genes at each step. Reasons for discarding genes are provided at each step. Proportions of genes with no reads, or with marginal expression, and obtained after filtering are shown by time point in C

B, Read assignment by category per sample. Reads are categorized as "Unmapped" where they could not be assigned, "MultiMap", where they mapped to multiple loci, "Ambiguity", where they spanned multiple genomic features, "No Feature" where they did not overlap any genomic feature, and "Genes" where reads could be mapped to single genomic features, and were taken further.

C, Proportions of genes with no reads, or with marginal expression, and obtained after filtering and used for used for between-group comparisons are shown at each time point.
