# Supplementary material for: Transcriptomic Analysis of Inflammatory Cardiomyopathy Identifies Molecular Signatures of Disease and Informs in silico Prediction of a Network-Based Rationale for Therapy
Source: Front Immunol. 2021 Mar 5;12:640837. doi: 10.3389/fimmu.2021.640837 (PMC7973371; doi:10.3389/fimmu.2021.640837)
Supplement: Supplementary file 2 [file Data_Sheet_2.zip › Myocarditis/gene-subnetwork-analysis.html]

Chapter 6 Gene subnetwork analysis | Combinatorial attack on a gene subnetwork during experimental autoimmune myocarditis


- Myocarditis
- **1** Overview
- **2** QC and differential analysis
- **3** List of differential genes
- **4** Gene groupings
  - **4.1** R function Upset
  - **4.2** Group visualisation
  - **4.3** Heatmap visualisation
- **5** Pathway analysis
  - **5.1** Enrichment analysis
  - **5.2** Enriched pathways
- **6** Gene subnetwork analysis
  - **6.1** Subnetwork identification
  - **6.2** Gene nodes in the subnetwork
  - **6.3** Interacting edges in the subnetwork
  - **6.4** Subnetwork visualisation
- **7** Combinatorial attack
  - **7.1** R function CombAttack
  - **7.2** Individual nodes
  - **7.3** Two-node combination
- **8** Session Info

# Combinatorial attack on a gene subnetwork during experimental autoimmune myocarditis

# Chapter 6 Gene subnetwork analysis

We perform an integrated analysis of KEGG molecular interactions with gene expression changes at different time points and identified a subnetwork of 50 gene nodes based on their expression importance over time. The cascade of subnetwork activation is visualised through the course of the disease process, where fold change over time and differential significance of individual genes is indicated by color and size respectively of individual gene nodes. For details, please refer to our paper.
