# Supplementary material for: Transcriptomic Analysis of Inflammatory Cardiomyopathy Identifies Molecular Signatures of Disease and Informs in silico Prediction of a Network-Based Rationale for Therapy
Source: Front Immunol. 2021 Mar 5;12:640837. doi: 10.3389/fimmu.2021.640837 (PMC7973371; doi:10.3389/fimmu.2021.640837)
Supplement: Supplementary file 2 [file Data_Sheet_2.zip › Myocarditis/index.html]

Identification of and combinatorial attack on a gene subnetwork active during experimental autoimmune myocarditis


- Myocarditis
- **1** Overview
- **2** RNAseq analysis (quality control and differential analysis)
- **3** List of differentially expressed genes
- **4** R packages required
- **5** Gene groupings
  - **5.1** R function Upset
  - **5.2** Group visualisation
  - **5.3** Grouped genes
  - **5.4** Heatmap visualisation
- **6** Pathway analysis
  - **6.1** Enrichment analysis
  - **6.2** Enriched pathways
- **7** Subnetwork analysis
  - **7.1** Subnetwork identification
  - **7.2** Subnetwork visualisation
  - **7.3** Gene nodes in the subnetwork
  - **7.4** Edges in the subnetwork
- **8** Combinatorial attack analysis
  - **8.1** R function CombAttack
  - **8.2** Individual nodes
  - **8.3** Two-node combination
- **9** R session information
- **10** Flow cytometry data

# Identification of and combinatorial attack on a gene subnetwork active during experimental autoimmune myocarditis

# Identification of and combinatorial attack on a gene subnetwork active during experimental autoimmune myocarditis

*Wellcome Centre for Human Genetics, University of Oxford*

*2020-10-12*

# Chapter 1 Overview

In this website, we provide information including inputs (such as List of differentially expressed genes), outputs (such as Grouped genes, Enriched pathways, Gene nodes in the subnetwork and Edges in the subnetwork), functions (such as R function CombAttack) and many others necessary to perform Pathway analysis, Subnetwork analysis and Combinatorial attack analysis. In particular, we introduce a new metric `attackness` to quantify the tolerance of the network to individual node removal, defined as the fraction of network nodes disconnected from the giant component after node removal. The attackness ranges from 0 to 1, with the higher value indicating the more vulnerable (critical) node for the network. Similarly, we introduce a new concept `combinatorial attack` to maximise the attackness for nodes in a specific combination removed, for example, identifying the optimal combination involving any two nodes. Please email us for greater details and for the use analysing your own datasets.
