# Supplementary material for: Transcriptomic Analysis of Inflammatory Cardiomyopathy Identifies Molecular Signatures of Disease and Informs in silico Prediction of a Network-Based Rationale for Therapy
Source: Front Immunol. 2021 Mar 5;12:640837. doi: 10.3389/fimmu.2021.640837 (PMC7973371; doi:10.3389/fimmu.2021.640837)
Supplement: Supplementary file 2 [file Data_Sheet_2.zip › Myocarditis/subnetwork-visualisation.html]

7.2 Subnetwork visualisation | Identification of and combinatorial attack on a gene subnetwork active during experimental autoimmune myocarditis


- Myocarditis
- **1** Overview
- **2** RNAseq analysis (quality control and differential analysis)
- **3** List of differentially expressed genes
- **4** R packages required
- **5** Gene groupings
  - **5.1** R function Upset
  - **5.2** Group visualisation
  - **5.3** Grouped genes
  - **5.4** Heatmap visualisation
- **6** Pathway analysis
  - **6.1** Enrichment analysis
  - **6.2** Enriched pathways
- **7** Subnetwork analysis
  - **7.1** Subnetwork identification
  - **7.2** Subnetwork visualisation
  - **7.3** Gene nodes in the subnetwork
  - **7.4** Edges in the subnetwork
- **8** Combinatorial attack analysis
  - **8.1** R function CombAttack
  - **8.2** Individual nodes
  - **8.3** Two-node combination
- **9** R session information
- **10** Flow cytometry data

# Identification of and combinatorial attack on a gene subnetwork active during experimental autoimmune myocarditis

## 7.2 Subnetwork visualisation

In the context of the whole/parent network (`ig`), visualise the gene subnetwork (`subg`), with 50 gene nodes labelled by gene symbols and 75 connections between nodes (network edges).

```
library(XGR)

## "ig" (the parent network) marked by the "subg" (the subnetwork)
ig_marked <- xMarkNet(ig, subg)

## the object "ig_marked" appended with two node attributes "xcoord" and "ycoord"
ig_marked %>% xLayout("graphlayouts.layout_with_stress") -> ig_marked

## the object "ig_marked" appended with two edge attributes
## "color" for edge coloring and "color.alpha" for edge color transparency
E(ig_marked)$color <- ifelse(E(ig_marked)$mark==0, "grey90", "cyan4")
E(ig_marked)$color.alpha <- ifelse(E(ig_marked)$mark==0, 0.1, 0.3)
V(ig_marked)$label <- ifelse(V(ig_marked)$highlight==0, '', V(ig_marked)$name)

## visualise the parent network highlighted by the subnetwork
## nodes placed by coordinates
## nodes colored differently, thus, being highlighted
## edges colored differently, thus, being highlighted
gg_marked <- ig_marked %>% xGGnetwork(node.label='label', node.label.size=2.5, node.label.color='darkblue', node.label.alpha=0.8, node.label.padding=0.1, node.label.arrow=0, node.label.force=0.01, , node.xcoord="xcoord", node.ycoord="ycoord", node.color="highlight", colormap="grey60-cyan4", node.color.alpha=0.4, node.size='highlight', node.size.range=c(0.5,2), edge.color="color", edge.color.alpha="color.alpha",edge.curve=0,edge.arrow.gap=0.01) + guides(color="none", size="none")
gg_marked
```

Gene subnetwork illustrated at 3 different time-points with the same layout as shown above. Nodes at each time point are coloured by log2(fold change) and sized by -log10(FDR) as indicated in the scale.

```
library(XGR)

## the object "subg" (subnetwork) appended with two node attributes "xcoord" and "ycoord"
ind <- match(V(subg)$name, V(ig_marked)$name)
V(subg)$xcoord <- V(ig_marked)$xcoord[ind]
V(subg)$ycoord <- V(ig_marked)$ycoord[ind]

## do visualisation
ls_subg <- lapply(seq(ncol(mat_FC)), function(j){
    g_tmp <- subg
    ind <- match(V(g_tmp)$name, rownames(mat_FC))
    V(g_tmp)$logFC <- mat_FC[ind,j]
    ind <- match(V(g_tmp)$name, rownames(mat_FDR))
    V(g_tmp)$logFDR <- -log10(mat_FDR[ind,j])
    g_tmp
})
names(ls_subg) <- colnames(mat_FC)
gg_timepoints <- xGGnetwork(ls_subg, node.label='', node.label.size=2.5, node.label.color='darkblue', node.label.alpha=0.8, node.label.padding=0.1, node.label.arrow=0, node.label.force=0.05, node.shape=19, node.xcoord='xcoord', node.ycoord='ycoord', node.color='logFC', node.color.title=expression(log[2]("FC")), colormap=c("darkblue-lightyellow-darkred",'jet.top','lightyellow-orange-darkred',"brewer.YlOrRd","ggplot2")[1], ncolors=64, zlim=c(-4,4), node.size='logFDR', node.size.title=expression(-log[10]("FDR")), node.size.range=c(0,2), slim=c(0,20), edge.color="cyan4",edge.color.alpha=0.2,edge.curve=0,edge.arrow.gap=0, title='', ncolumns=3)
gg_timepoints
```
