# Supplementary material for: Transcriptomic Analysis of Inflammatory Cardiomyopathy Identifies Molecular Signatures of Disease and Informs in silico Prediction of a Network-Based Rationale for Therapy
Source: Front Immunol. 2021 Mar 5;12:640837. doi: 10.3389/fimmu.2021.640837 (PMC7973371; doi:10.3389/fimmu.2021.640837)
Supplement: Supplementary file 2 [file Data_Sheet_2.zip › Myocarditis/individual-nodes.html]

8.2 Individual nodes | Identification of and combinatorial attack on a gene subnetwork active during experimental autoimmune myocarditis


- Myocarditis
- **1** Overview
- **2** RNAseq analysis (quality control and differential analysis)
- **3** List of differentially expressed genes
- **4** R packages required
- **5** Gene groupings
  - **5.1** R function Upset
  - **5.2** Group visualisation
  - **5.3** Grouped genes
  - **5.4** Heatmap visualisation
- **6** Pathway analysis
  - **6.1** Enrichment analysis
  - **6.2** Enriched pathways
- **7** Subnetwork analysis
  - **7.1** Subnetwork identification
  - **7.2** Subnetwork visualisation
  - **7.3** Gene nodes in the subnetwork
  - **7.4** Edges in the subnetwork
- **8** Combinatorial attack analysis
  - **8.1** R function CombAttack
  - **8.2** Individual nodes
  - **8.3** Two-node combination
- **9** R session information
- **10** Flow cytometry data

# Identification of and combinatorial attack on a gene subnetwork active during experimental autoimmune myocarditis

## 8.2 Individual nodes

Here we illustrate how to calculate attackness for each node in the 50-gene subnetwork (see `subg` above). Independent of previous steps, this subnetwork (`subg`) can be also imported from subnetwork\_node\_info.txt and subnetwork\_edge\_info.txt; see below.

```
library(tidyverse)
library(igraph)
RData.location <- "http://galahad.well.ox.ac.uk/Myocarditis"
```

```
## ig: 50-gene subnetwork
df_nodes <- str_c(RData.location, '/subnetwork_node_info.txt') %>% read_delim(delim="\t") %>% select(name, description)
df_edges <- str_c(RData.location, '/subnetwork_edge_info.txt') %>% read_delim(delim="\t")
subg <- igraph::graph_from_data_frame(d=df_edges, directed=F, vertices=df_nodes)

## attackness for each node
df_nodes %>% pull(name) %>% utils::combn(1,simplify=F) -> combine
attackness <- CombAttack(subg, combine)

## append 'attackness' to df_nodes
df_nodes %>% inner_join(attackness %>% transmute(name=nodes.removed, attackness=frac.disconnected), by='name') %>% arrange(desc(attackness)) -> df_nodes
```

The results are stored in a tibble called `df_nodes`, a data frame with three columns `name`, `description` and `attackness` (sorted in a descending order).

```
df_nodes
## # A tibble: 50 x 3
##    name     description                                               attackness
##    <chr>    <chr>                                                          <dbl>
##  1 Traf2    TNF receptor-associated factor 2                                0.5 
##  2 Nfkb1    nuclear factor of kappa light polypeptide gene enhancer …       0.42
##  3 Ccl5     chemokine (C-C motif) ligand 5                                  0.18
##  4 Rac1     Rac family small GTPase 1                                       0.14
##  5 Vav1     vav 1 oncogene                                                  0.06
##  6 Vcam1    vascular cell adhesion molecule 1                               0.06
##  7 Ncf1     neutrophil cytosolic factor 1                                   0.04
##  8 Tnfrsf1b tumor necrosis factor receptor superfamily, member 1b           0.04
##  9 Lck      lymphocyte protein tyrosine kinase                              0.04
## 10 Gsdmd    gasdermin D                                                     0.04
## # … with 40 more rows
```
