# Supplementary material for: Transcriptomic Analysis of Inflammatory Cardiomyopathy Identifies Molecular Signatures of Disease and Informs in silico Prediction of a Network-Based Rationale for Therapy
Source: Front Immunol. 2021 Mar 5;12:640837. doi: 10.3389/fimmu.2021.640837 (PMC7973371; doi:10.3389/fimmu.2021.640837)
Supplement: Supplementary file 2 [file Data_Sheet_2.zip › Myocarditis/interacting-edges.html]

4.3 Interacting edges | Combinatorial attack on a gene subnetwork during experimental autoimmune myocarditis


- Myocarditis
- **1** Overview
- **2** Differential genes
  - **2.1** QC and differential analysis
  - **2.2** List of differential genes
  - **2.3** Gene groupings
- **3** Pathway enrichment analysis
  - **3.1** Enrichment analysis
  - **3.2** Enriched pathways
- **4** Gene subnetwork analysis
  - **4.1** Network analysis
  - **4.2** Gene nodes
  - **4.3** Interacting edges
  - **4.4** Network visualisation
- **5** Combinatorial attack
  - **5.1** R function CombAttack
  - **5.2** Individual nodes
  - **5.3** Two-node combination
- **6** Session Info

# Combinatorial attack on a gene subnetwork during experimental autoimmune myocarditis

## 4.3 Interacting edges

Available at subnetwork\_edge\_info.txt or below.
