# Supplementary material for: Transcriptomic Analysis of Inflammatory Cardiomyopathy Identifies Molecular Signatures of Disease and Informs in silico Prediction of a Network-Based Rationale for Therapy
Source: Front Immunol. 2021 Mar 5;12:640837. doi: 10.3389/fimmu.2021.640837 (PMC7973371; doi:10.3389/fimmu.2021.640837)
Supplement: Supplementary file 2 [file Data_Sheet_2.zip › Myocarditis/two-node-combination.html]

8.3 Two-node combination | Identification of and combinatorial attack on a gene subnetwork active during experimental autoimmune myocarditis


- Myocarditis
- **1** Overview
- **2** RNAseq analysis (quality control and differential analysis)
- **3** List of differentially expressed genes
- **4** R packages required
- **5** Gene groupings
  - **5.1** R function Upset
  - **5.2** Group visualisation
  - **5.3** Grouped genes
  - **5.4** Heatmap visualisation
- **6** Pathway analysis
  - **6.1** Enrichment analysis
  - **6.2** Enriched pathways
- **7** Subnetwork analysis
  - **7.1** Subnetwork identification
  - **7.2** Subnetwork visualisation
  - **7.3** Gene nodes in the subnetwork
  - **7.4** Edges in the subnetwork
- **8** Combinatorial attack analysis
  - **8.1** R function CombAttack
  - **8.2** Individual nodes
  - **8.3** Two-node combination
- **9** R session information
- **10** Flow cytometry data

# Identification of and combinatorial attack on a gene subnetwork active during experimental autoimmune myocarditis

## 8.3 Two-node combination

As an exemplar for combinatorial attack, we here remove any two nodes in combination, and assess the effect of such removal on the fraction of network nodes disconnected from the giant component.

```
df_nodes %>% arrange(-attackness) %>% pull(name) %>% utils::combn(2, simplify=F) -> combine
df_combine <- CombAttack(subg, combine) %>% transmute(nodes.removed=nodes.removed, attackness=frac.disconnected) %>% arrange(-attackness)
```

The results are stored in a tibble called `df_combine`, a data frame with two columns `nodes.removed` (removed nodes in combination) and `attackness` (sorted in a descending order).

```
df_combine
## # A tibble: 1,225 x 2
##    nodes.removed attackness
##    <chr>              <dbl>
##  1 Rac1,Traf2          0.56
##  2 Vav1,Traf2          0.56
##  3 Pik3cd,Traf2        0.56
##  4 Rac1,Nfkb1          0.56
##  5 Ncf1,Traf2          0.54
##  6 Traf2,Lck           0.54
##  7 Traf2,Cyba          0.54
##  8 Fgr,Traf2           0.52
##  9 Hck,Traf2           0.52
## 10 Prkcd,Traf2         0.52
## # … with 1,215 more rows
```
