# Supplementary material for: Transcriptomic Analysis of Inflammatory Cardiomyopathy Identifies Molecular Signatures of Disease and Informs in silico Prediction of a Network-Based Rationale for Therapy
Source: Front Immunol. 2021 Mar 5;12:640837. doi: 10.3389/fimmu.2021.640837 (PMC7973371; doi:10.3389/fimmu.2021.640837)
Supplement: Supplementary file 2 [file Data_Sheet_2.zip › Myocarditis/rnaseq-analysis-quality-control-and-differential-analysis.html]

Chapter 2 RNAseq analysis (quality control and differential analysis) | Identification of and combinatorial attack on a gene subnetwork active during experimental autoimmune myocarditis


- Myocarditis
- **1** Overview
- **2** RNAseq analysis (quality control and differential analysis)
- **3** List of differentially expressed genes
- **4** R packages required
- **5** Gene groupings
  - **5.1** R function Upset
  - **5.2** Group visualisation
  - **5.3** Grouped genes
  - **5.4** Heatmap visualisation
- **6** Pathway analysis
  - **6.1** Enrichment analysis
  - **6.2** Enriched pathways
- **7** Subnetwork analysis
  - **7.1** Subnetwork identification
  - **7.2** Subnetwork visualisation
  - **7.3** Gene nodes in the subnetwork
  - **7.4** Edges in the subnetwork
- **8** Combinatorial attack analysis
  - **8.1** R function CombAttack
  - **8.2** Individual nodes
  - **8.3** Two-node combination
- **9** R session information
- **10** Flow cytometry data

# Identification of and combinatorial attack on a gene subnetwork active during experimental autoimmune myocarditis

# Chapter 2 RNAseq analysis (quality control and differential analysis)

Instructions on performing QC and differential analysis can be downloaded here.

```
README.txt
P180748_P180481_secondary_QC.pdf
RNA_QC.R
analysis_performed.R
edgeR_analysis.R
```

A zip folder containing the working RNAseq analysis scripts with metadata, and example analysis of the D21 myocarditis vs D21control analysis can be found at here

Information above is provided from Bioinformatics Core at Wellcome Centre for Human Genetics, University of Oxford; please contain Helen for details.
