# Supplementary material for: Transcriptomic Analysis of Inflammatory Cardiomyopathy Identifies Molecular Signatures of Disease and Informs in silico Prediction of a Network-Based Rationale for Therapy
Source: Front Immunol. 2021 Mar 5;12:640837. doi: 10.3389/fimmu.2021.640837 (PMC7973371; doi:10.3389/fimmu.2021.640837)
Supplement: Supplementary file 2 [file Data_Sheet_2.zip › Myocarditis/r-function-upset.html]

5.1 R function Upset | Identification of and combinatorial attack on a gene subnetwork active during experimental autoimmune myocarditis


- Myocarditis
- **1** Overview
- **2** RNAseq analysis (quality control and differential analysis)
- **3** List of differentially expressed genes
- **4** R packages required
- **5** Gene groupings
  - **5.1** R function Upset
  - **5.2** Group visualisation
  - **5.3** Grouped genes
  - **5.4** Heatmap visualisation
- **6** Pathway analysis
  - **6.1** Enrichment analysis
  - **6.2** Enriched pathways
- **7** Subnetwork analysis
  - **7.1** Subnetwork identification
  - **7.2** Subnetwork visualisation
  - **7.3** Gene nodes in the subnetwork
  - **7.4** Edges in the subnetwork
- **8** Combinatorial attack analysis
  - **8.1** R function CombAttack
  - **8.2** Individual nodes
  - **8.3** Two-node combination
- **9** R session information
- **10** Flow cytometry data

# Identification of and combinatorial attack on a gene subnetwork active during experimental autoimmune myocarditis

## 5.1 R function Upset

We prepare an R function called `Upset` to visualise gene groups.

```
## mat: a binary matrix with row names for gene members and column names for elementary components (eg time-specific up-regulated or down-regulated genes)
## min.freq: the minumum frequency/number allowed per combination/group (30 by default)
Upset <- function(mat, min.freq=30)
{
    
    ## a vector of members (with the same order as provided)
    levels <- colnames(mat)

    ## code_full
    mat %>% tibble::as_tibble(rownames='member') %>% tidyr::unite(code,-member,sep='-',remove=FALSE) %>% dplyr::mutate(ncode=dplyr::select(.,c(-member,-code)) %>% base::rowSums()) -> df
    df %>% dplyr::inner_join(df %>% dplyr::count(code,name='freq'), by='code') %>% arrange(-ncode,-freq,desc(code)) -> code_full
    
    ## ggplot
    code_full %>% dplyr::select(-member) %>% dplyr::distinct() %>% tidyr::pivot_longer(cols=c(-code,-ncode,-freq),names_to='name', values_to='value') %>% dplyr::filter(value==1) %>% dplyr::select(-value) %>% dplyr::group_by(code,ncode,freq) %>% dplyr::summarise(data=stringr::str_c(name,collapse=',')) %>% dplyr::ungroup() %>% dplyr::arrange(ncode,freq) -> df
    if(!is.null(min.freq)){
        df %>% dplyr::filter(freq>=min.freq) -> df
    }
    df %>% dplyr::mutate(data=forcats::fct_inorder(data)) %>% ggplot(aes(x=data,y=freq)) + geom_col(fill="steelblue", color='transparent', width=0.5, alpha=0.8) + theme_classic() + ggupset::axis_combmatrix(sep=",", levels=levels) -> gp
    
    gp <- gp + ggupset::theme_combmatrix(combmatrix.panel.point.color.fill="steelblue", combmatrix.panel.point.color.empty="grey90", combmatrix.panel.point.size=1.5, combmatrix.panel.line.size=0.1)
    gp <- gp + theme(axis.title.x=element_blank()) + ylab('Number of genes\nper group')
    
    ## gp$full
    gp$full <- code_full %>% tidyr::nest(data=member) %>% dplyr::mutate(member=purrr::map_chr(data,~stringr::str_c(.x %>% dplyr::pull(member), collapse=','))) %>% dplyr::select(-data) %>% dplyr::arrange(-ncode,-freq,desc(code)) %>% tidyr::separate_rows(member, sep=',')
    
    return(gp)
}
```
