# Supplementary material for: Transcriptomic Analysis of Inflammatory Cardiomyopathy Identifies Molecular Signatures of Disease and Informs in silico Prediction of a Network-Based Rationale for Therapy
Source: Front Immunol. 2021 Mar 5;12:640837. doi: 10.3389/fimmu.2021.640837 (PMC7973371; doi:10.3389/fimmu.2021.640837)
Supplement: Supplementary file 2 [file Data_Sheet_2.zip › Myocarditis/pathway-enrichment-analysis.html]

Chapter 3 Pathway enrichment analysis | Combinatorial attack on a gene subnetwork during experimental autoimmune myocarditis


- Myocarditis
- **1** Overview
- **2** Differential genes
  - **2.1** QC and differential analysis
  - **2.2** List of differential genes
  - **2.3** Gene groupings
- **3** Pathway enrichment analysis
  - **3.1** Enrichment analysis
  - **3.2** Enriched pathways
- **4** Gene subnetwork analysis
  - **4.1** Network analysis
  - **4.2** Gene nodes
  - **4.3** Interacting edges
  - **4.4** Network visualisation
- **5** Combinatorial attack
  - **5.1** R function CombAttack
  - **5.2** Individual nodes
  - **5.3** Two-node combination
- **6** Session Info

# Combinatorial attack on a gene subnetwork during experimental autoimmune myocarditis

# Chapter 3 Pathway enrichment analysis

Gene groups identified above (see `df_full`) are subjected to pathway enrichment analysis using the XGR package. Pathways and member genes (mouse) are obtained from KEGG (accessed on June 2020). Such pathway enrichment analysis is carried out for KEGG Organismal Systems pathways and Environmental Information Processing pathways.

Gene group codes and abbreviations are as follows: `1-0-1-0-1-0` for early-persistent induced (`EPi`); `0-0-1-0-1-0` for mid-persistent induced (`MPi`); `0-0-0-1-0-1` for mid-persistent repressed (`MPr`); `0-0-1-0-0-0` for mid-transient induced (`MTi`); `0-0-0-1-0-0` for mid-transient repressed (`MTr`); `0-0-0-0-1-0` for late-transient induced (`LTi`); and `0-0-0-0-0-1` for late-transient repressed (`LTr`).
