# Supplementary material for: Transcriptomic Analysis of Inflammatory Cardiomyopathy Identifies Molecular Signatures of Disease and Informs in silico Prediction of a Network-Based Rationale for Therapy
Source: Front Immunol. 2021 Mar 5;12:640837. doi: 10.3389/fimmu.2021.640837 (PMC7973371; doi:10.3389/fimmu.2021.640837)
Supplement: Supplementary file 2 [file Data_Sheet_2.zip › Myocarditis/heatmap-visualisation.html]

5.4 Heatmap visualisation | Identification of and combinatorial attack on a gene subnetwork active during experimental autoimmune myocarditis


- Myocarditis
- **1** Overview
- **2** RNAseq analysis (quality control and differential analysis)
- **3** List of differentially expressed genes
- **4** R packages required
- **5** Gene groupings
  - **5.1** R function Upset
  - **5.2** Group visualisation
  - **5.3** Grouped genes
  - **5.4** Heatmap visualisation
- **6** Pathway analysis
  - **6.1** Enrichment analysis
  - **6.2** Enriched pathways
- **7** Subnetwork analysis
  - **7.1** Subnetwork identification
  - **7.2** Subnetwork visualisation
  - **7.3** Gene nodes in the subnetwork
  - **7.4** Edges in the subnetwork
- **8** Combinatorial attack analysis
  - **8.1** R function CombAttack
  - **8.2** Individual nodes
  - **8.3** Two-node combination
- **9** R session information
- **10** Flow cytometry data

# Identification of and combinatorial attack on a gene subnetwork active during experimental autoimmune myocarditis

## 5.4 Heatmap visualisation

Visualise, for example, genes with the code `1-0-1-0-1-0` or early-persistent induced (`EPi`).

```
library(XGR)

gp_triple <- read_delim('DE_genes.txt.gz', delim='\t') %>% semi_join(df_full %>% filter(code=='1-0-1-0-1-0'),by=c('mgi_symbol'='member')) %>% select(mgi_symbol,time,logFC) %>% pivot_wider(names_from=time, values_from=logFC) %>% column_to_rownames('mgi_symbol') %>% xHeatmap(reorder="row", colormap="darkblue-lightyellow-darkred", zlim=c(-4,4), x.rotate=0, shape='tile', x.text.size=8, y.text.size=0, barwidth=0.8, barheight=5)
gp_triple
```
