# Supplementary material for: Transcriptomic Analysis of Inflammatory Cardiomyopathy Identifies Molecular Signatures of Disease and Informs in silico Prediction of a Network-Based Rationale for Therapy
Source: Front Immunol. 2021 Mar 5;12:640837. doi: 10.3389/fimmu.2021.640837 (PMC7973371; doi:10.3389/fimmu.2021.640837)
Supplement: Supplementary file 2 [file Data_Sheet_2.zip › Myocarditis/r-session-information.html]

Chapter 9 R session information | Identification of and combinatorial attack on a gene subnetwork active during experimental autoimmune myocarditis


- Myocarditis
- **1** Overview
- **2** RNAseq analysis (quality control and differential analysis)
- **3** List of differentially expressed genes
- **4** R packages required
- **5** Gene groupings
  - **5.1** R function Upset
  - **5.2** Group visualisation
  - **5.3** Grouped genes
  - **5.4** Heatmap visualisation
- **6** Pathway analysis
  - **6.1** Enrichment analysis
  - **6.2** Enriched pathways
- **7** Subnetwork analysis
  - **7.1** Subnetwork identification
  - **7.2** Subnetwork visualisation
  - **7.3** Gene nodes in the subnetwork
  - **7.4** Edges in the subnetwork
- **8** Combinatorial attack analysis
  - **8.1** R function CombAttack
  - **8.2** Individual nodes
  - **8.3** Two-node combination
- **9** R session information
- **10** Flow cytometry data

# Identification of and combinatorial attack on a gene subnetwork active during experimental autoimmune myocarditis

# Chapter 9 R session information

Table 9.1: Session info about R, the OS, and attached/loaded packages


| pkg | version | title |
| --- | --- | --- |
| dnet | 1.1.7 | Integrative Analysis of Omics Data in Terms of Network, Evolution and Ontology |
| dplyr | 1.0.1 | A Grammar of Data Manipulation |
| forcats | 0.5.0 | Tools for Working with Categorical Variables (Factors) |
| ggplot2 | 3.3.2 | Create Elegant Data Visualisations Using the Grammar of Graphics |
| ggupset | 0.3.0 | Combination Matrix Axis for 'ggplot2' to Create 'UpSet' Plots |
| hexbin | 1.28.1 | Hexagonal Binning Routines |
| igraph | 1.2.5 | Network Analysis and Visualization |
| png | 0.1-7 | Read and write PNG images |
| purrr | 0.3.4 | Functional Programming Tools |
| readr | 1.3.1 | Read Rectangular Text Data |
| stringr | 1.4.0 | Simple, Consistent Wrappers for Common String Operations |
| supraHex | 1.27.3 | supraHex: a supra-hexagonal map for analysing tabular omics data |
| tibble | 3.0.3 | Simple Data Frames |
| tidyr | 1.1.1 | Tidy Messy Data |
| tidyverse | 1.3.0 | Easily Install and Load the 'Tidyverse' |
| XGR | 1.1.8 | Exploring Genomic Relations for Enhanced Interpretation Through Enrichment, Similarity, Network and Annotation Analysis |
| ape | 5.4 | Analyses of Phylogenetics and Evolution |
| assertthat | 0.2.1 | Easy Pre and Post Assertions |
| backports | 1.1.8 | Reimplementations of Functions Introduced Since R-3.0.0 |
| BiocGenerics | 0.34.0 | S4 generic functions used in Bioconductor |
| bitops | 1.0-6 | Bitwise Operations |
| blob | 1.2.1 | A Simple S3 Class for Representing Vectors of Binary Data ('BLOBS') |
| bookdown | 0.20 | Authoring Books and Technical Documents with R Markdown |
| broom | 0.7.0 | Convert Statistical Objects into Tidy Tibbles |
| cellranger | 1.1.0 | Translate Spreadsheet Cell Ranges to Rows and Columns |
| cli | 2.0.2 | Helpers for Developing Command Line Interfaces |
| colorspace | 1.4-1 | A Toolbox for Manipulating and Assessing Colors and Palettes |
| compiler | 4.0.2 | The R Compiler Package |
| crayon | 1.3.4 | Colored Terminal Output |
| crosstalk | 1.1.0.1 | Inter-Widget Interactivity for HTML Widgets |
| crul | 1.0.0 | HTTP Client |
| curl | 4.3 | A Modern and Flexible Web Client for R |
| DBI | 1.1.0 | R Database Interface |
| dbplyr | 1.4.4 | A 'dplyr' Back End for Databases |
| digest | 0.6.25 | Create Compact Hash Digests of R Objects |
| DT | 0.14 | A Wrapper of the JavaScript Library 'DataTables' |
| ellipsis | 0.3.1 | Tools for Working with ... |
| evaluate | 0.14 | Parsing and Evaluation Tools that Provide More Details than the Default |
| fansi | 0.4.1 | ANSI Control Sequence Aware String Functions |
| farver | 2.0.3 | High Performance Colour Space Manipulation |
| fs | 1.5.0 | Cross-Platform File System Operations Based on 'libuv' |
| generics | 0.0.2 | Common S3 Generics not Provided by Base R Methods Related to Model Fitting |
| GenomeInfoDb | 1.24.2 | Utilities for manipulating chromosome names, including modifying them to follow a particular naming style |
| GenomeInfoDbData | 1.2.3 | Species and taxonomy ID look up tables used by GenomeInfoDb |
| GenomicRanges | 1.40.0 | Representation and manipulation of genomic intervals |
| ggnetwork | 0.5.8 | Geometries to Plot Networks with 'ggplot2' |
| ggrepel | 0.8.2 | Automatically Position Non-Overlapping Text Labels with 'ggplot2' |
| glue | 1.4.1 | Interpreted String Literals |
| graph | 1.66.0 | graph: A package to handle graph data structures |
| graphlayouts | 0.7.0 | Additional Layout Algorithms for Network Visualizations |
| gtable | 0.3.0 | Arrange 'Grobs' in Tables |
| haven | 2.3.1 | Import and Export 'SPSS', 'Stata' and 'SAS' Files |
| highr | 0.8 | Syntax Highlighting for R Source Code |
| hms | 0.5.3 | Pretty Time of Day |
| htmltools | 0.5.0 | Tools for HTML |
| htmlwidgets | 1.5.1 | HTML Widgets for R |
| httpcode | 0.3.0 | 'HTTP' Status Code Helper |
| httr | 1.4.2 | Tools for Working with URLs and HTTP |
| IRanges | 2.22.2 | Foundation of integer range manipulation in Bioconductor |
| jsonlite | 1.7.0 | A Robust, High Performance JSON Parser and Generator for R |
| knitr | 1.29 | A General-Purpose Package for Dynamic Report Generation in R |
| labeling | 0.3 | Axis Labeling |
| lattice | 0.20-41 | Trellis Graphics for R |
| lifecycle | 0.2.0 | Manage the Life Cycle of your Package Functions |
| lubridate | 1.7.9 | Make Dealing with Dates a Little Easier |
| magrittr | 1.5 | A Forward-Pipe Operator for R |
| MASS | 7.3-51.6 | Support Functions and Datasets for Venables and Ripley's MASS |
| Matrix | 1.2-18 | Sparse and Dense Matrix Classes and Methods |
| memoise | 1.1.0 | Memoisation of Functions |
| modelr | 0.1.8 | Modelling Functions that Work with the Pipe |
| munsell | 0.5.0 | Utilities for Using Munsell Colours |
| nlme | 3.1-148 | Linear and Nonlinear Mixed Effects Models |
| osfr | 0.2.8 | Interface to the 'Open Science Framework' ('OSF') |
| parallel | 4.0.2 | Support for Parallel computation in R |
| pillar | 1.4.6 | Coloured Formatting for Columns |
| pkgconfig | 2.0.3 | Private Configuration for 'R' Packages |
| R6 | 2.4.1 | Encapsulated Classes with Reference Semantics |
| RCircos | 1.2.1 | Circos 2D Track Plot |
| Rcpp | 1.0.5 | Seamless R and C++ Integration |
| RCurl | 1.98-1.2 | General Network (HTTP/FTP/...) Client Interface for R |
| readxl | 1.3.1 | Read Excel Files |
| reprex | 0.3.0 | Prepare Reproducible Example Code via the Clipboard |
| Rgraphviz | 2.32.0 | Provides plotting capabilities for R graph objects |
| rlang | 0.4.7 | Functions for Base Types and Core R and 'Tidyverse' Features |
| rmarkdown | 2.3 | Dynamic Documents for R |
| rstudioapi | 0.11 | Safely Access the RStudio API |
| rvest | 0.3.6 | Easily Harvest (Scrape) Web Pages |
| S4Vectors | 0.26.1 | Foundation of vector-like and list-like containers in Bioconductor |
| scales | 1.1.1 | Scale Functions for Visualization |
| stats4 | 4.0.2 | Statistical Functions using S4 Classes |
| stringi | 1.4.6 | Character String Processing Facilities |
| tidyselect | 1.1.0 | Select from a Set of Strings |
| tools | 4.0.2 | Tools for Package Development |
| utf8 | 1.1.4 | Unicode Text Processing |
| vctrs | 0.3.2 | Vector Helpers |
| withr | 2.2.0 | Run Code 'With' Temporarily Modified Global State |
| xfun | 0.16 | Miscellaneous Functions by 'Yihui Xie' |
| xml2 | 1.3.2 | Parse XML |
| XVector | 0.28.0 | Foundation of external vector representation and manipulation in Bioconductor |
| yaml | 2.2.1 | Methods to Convert R Data to YAML and Back |
| zlibbioc | 1.34.0 | An R packaged zlib-1.2.5 |
