# Supplementary material for: Transcriptomic Analysis of Inflammatory Cardiomyopathy Identifies Molecular Signatures of Disease and Informs in silico Prediction of a Network-Based Rationale for Therapy
Source: Front Immunol. 2021 Mar 5;12:640837. doi: 10.3389/fimmu.2021.640837 (PMC7973371; doi:10.3389/fimmu.2021.640837)
Supplement: Supplementary file 2 [file Data_Sheet_2.zip › Myocarditis/group-visualisation.html]

5.2 Group visualisation | Identification of and combinatorial attack on a gene subnetwork active during experimental autoimmune myocarditis


- Myocarditis
- **1** Overview
- **2** RNAseq analysis (quality control and differential analysis)
- **3** List of differentially expressed genes
- **4** R packages required
- **5** Gene groupings
  - **5.1** R function Upset
  - **5.2** Group visualisation
  - **5.3** Grouped genes
  - **5.4** Heatmap visualisation
- **6** Pathway analysis
  - **6.1** Enrichment analysis
  - **6.2** Enriched pathways
- **7** Subnetwork analysis
  - **7.1** Subnetwork identification
  - **7.2** Subnetwork visualisation
  - **7.3** Gene nodes in the subnetwork
  - **7.4** Edges in the subnetwork
- **8** Combinatorial attack analysis
  - **8.1** R function CombAttack
  - **8.2** Individual nodes
  - **8.3** Two-node combination
- **9** R session information
- **10** Flow cytometry data

# Identification of and combinatorial attack on a gene subnetwork active during experimental autoimmune myocarditis

## 5.2 Group visualisation

```
library(tidyverse)
library(ggupset)

mat <- read_delim('DE_genes.txt.gz', delim='\t') %>% filter(group!='None') %>% arrange(time,desc(group)) %>% mutate(timegroup=str_c(time,'_',group)) %>% select(mgi_symbol, timegroup, logFC) %>% pivot_wider(names_from=timegroup, values_from=logFC, values_fill=list(logFC=0)) %>% column_to_rownames('mgi_symbol')
mat[mat!=0] <- 1

gp <- Upset(mat)
gp
```

Also, extract members per gene group stored in `df_full`. The same as `mat` above but with 4 columns added ('code', 'ncode', 'freq' and 'member').

```
df_full <- gp$full %>% filter(freq>=30)
df_full
## # A tibble: 4,433 x 10
##    code       D10_Up D10_Down D15_Up D15_Down D21_Up D21_Down ncode  freq member
##    <chr>       <dbl>    <dbl>  <dbl>    <dbl>  <dbl>    <dbl> <dbl> <int> <chr> 
##  1 1-0-1-0-1…      1        0      1        0      1        0     3   127 Il1a  
##  2 1-0-1-0-1…      1        0      1        0      1        0     3   127 Gm4841
##  3 1-0-1-0-1…      1        0      1        0      1        0     3   127 Cxcl9 
##  4 1-0-1-0-1…      1        0      1        0      1        0     3   127 Acod1 
##  5 1-0-1-0-1…      1        0      1        0      1        0     3   127 Cxcl10
##  6 1-0-1-0-1…      1        0      1        0      1        0     3   127 Socs3 
##  7 1-0-1-0-1…      1        0      1        0      1        0     3   127 Olr1  
##  8 1-0-1-0-1…      1        0      1        0      1        0     3   127 Gm121…
##  9 1-0-1-0-1…      1        0      1        0      1        0     3   127 Cxcl13
## 10 1-0-1-0-1…      1        0      1        0      1        0     3   127 Gm122…
## # … with 4,423 more rows
```

Write into a file 'Grouped\_genes.txt'

```
df_grouped_genes <- tibble(code=df_full %>% pull(code) %>% unique() %>% sort(), group=c('LTr','LTi','MTr','MPr','MTi','MPi','EPi')) %>% mutate(group=str_c(code,' (',group,')')) %>% inner_join(df_full, by='code') %>% select(-code,-ncode,-freq) 
df_grouped_genes %>% write_delim('Grouped_genes.txt',delim='\t')
```
