# Supplementary material for: Transcriptomic Analysis of Inflammatory Cardiomyopathy Identifies Molecular Signatures of Disease and Informs in silico Prediction of a Network-Based Rationale for Therapy
Source: Front Immunol. 2021 Mar 5;12:640837. doi: 10.3389/fimmu.2021.640837 (PMC7973371; doi:10.3389/fimmu.2021.640837)
Supplement: Supplementary file 2 [file Data_Sheet_2.zip › Myocarditis/gene-edges.html]

2.2 Gene edges | Combinatorial attack on a gene subnetwork during experimental autoimmune myocarditis


- Myocarditis
- **1** Overview
- **2** Gene subnetwork
  - **2.1** Gene nodes
  - **2.2** Gene edges
- **3** Combinatorial attack
  - **3.1** R function CombAttack
  - **3.2** Individual nodes
  - **3.3** Two-node combination
- **4** Session Info

# Combinatorial attack on a gene subnetwork during experimental autoimmune myocarditis

## 2.2 Gene edges
