# Supplementary material for: Transcriptomic Analysis of Inflammatory Cardiomyopathy Identifies Molecular Signatures of Disease and Informs in silico Prediction of a Network-Based Rationale for Therapy
Source: Front Immunol. 2021 Mar 5;12:640837. doi: 10.3389/fimmu.2021.640837 (PMC7973371; doi:10.3389/fimmu.2021.640837)
Supplement: Supplementary file 2 [file Data_Sheet_2.zip › Myocarditis/qc-and-differential-analysis.html]

Chapter 2 QC and differential analysis | Identification of and combinatorial attack on a gene subnetwork active during experimental autoimmune myocarditis


- Myocarditis
- **1** Overview
- **2** QC and differential analysis
- **3** List of differential genes
- **4** Getting ready
- **5** Gene groupings
  - **5.1** R function Upset
  - **5.2** Group visualisation
  - **5.3** Grouped genes
  - **5.4** Heatmap visualisation
- **6** Pathway analysis
  - **6.1** Enrichment analysis
  - **6.2** Enriched pathways
- **7** Subnetwork analysis
  - **7.1** Subnetwork identification
  - **7.2** Subnetwork visualisation
  - **7.3** Gene nodes in the subnetwork
  - **7.4** Edges in the subnetwork
- **8** Combinatorial attack
  - **8.1** R function CombAttack
  - **8.2** Individual nodes
  - **8.3** Two-node combination
- **9** Session Info
- **10** Flow cytometry data

# Identification of and combinatorial attack on a gene subnetwork active during experimental autoimmune myocarditis

# Chapter 2 QC and differential analysis

Instructions on performing QC and differential analysis can be downloaded here. Provided from Bioinformatics Core at Wellcome Centre for Human Genetics, University of Oxford; please contain Helen for details.

```
README.txt
P180748_P180481_secondary_QC.pdf
RNA_QC.R
analysis_performed.R
edgeR_analysis.R
```
