# Supplementary material for: Transcriptomic Analysis of Inflammatory Cardiomyopathy Identifies Molecular Signatures of Disease and Informs in silico Prediction of a Network-Based Rationale for Therapy
Source: Front Immunol. 2021 Mar 5;12:640837. doi: 10.3389/fimmu.2021.640837 (PMC7973371; doi:10.3389/fimmu.2021.640837)
Supplement: Supplementary file 2 [file Data_Sheet_2.zip › Myocarditis/interacting-edges-in-the-subnetwork.html]

6.3 Interacting edges in the subnetwork | Combinatorial attack on a gene subnetwork during experimental autoimmune myocarditis


- Myocarditis
- **1** Overview
- **2** QC and differential analysis
- **3** List of differential genes
- **4** Gene groupings
  - **4.1** R function Upset
  - **4.2** Group visualisation
  - **4.3** Heatmap visualisation
- **5** Pathway analysis
  - **5.1** Enrichment analysis
  - **5.2** Enriched pathways
- **6** Gene subnetwork analysis
  - **6.1** Subnetwork identification
  - **6.2** Gene nodes in the subnetwork
  - **6.3** Interacting edges in the subnetwork
  - **6.4** Subnetwork visualisation
- **7** Combinatorial attack
  - **7.1** R function CombAttack
  - **7.2** Individual nodes
  - **7.3** Two-node combination
- **8** Session Info

# Combinatorial attack on a gene subnetwork during experimental autoimmune myocarditis

## 6.3 Interacting edges in the subnetwork

Available at subnetwork\_edge\_info.txt or below.
