# Supplementary material for: Transcriptomic Analysis of Inflammatory Cardiomyopathy Identifies Molecular Signatures of Disease and Informs in silico Prediction of a Network-Based Rationale for Therapy
Source: Front Immunol. 2021 Mar 5;12:640837. doi: 10.3389/fimmu.2021.640837 (PMC7973371; doi:10.3389/fimmu.2021.640837)
Supplement: Supplementary file 2 [file Data_Sheet_2.zip › Myocarditis/r-function-combattack.html]

8.1 R function CombAttack | Identification of and combinatorial attack on a gene subnetwork active during experimental autoimmune myocarditis


- Myocarditis
- **1** Overview
- **2** RNAseq analysis (quality control and differential analysis)
- **3** List of differentially expressed genes
- **4** R packages required
- **5** Gene groupings
  - **5.1** R function Upset
  - **5.2** Group visualisation
  - **5.3** Grouped genes
  - **5.4** Heatmap visualisation
- **6** Pathway analysis
  - **6.1** Enrichment analysis
  - **6.2** Enriched pathways
- **7** Subnetwork analysis
  - **7.1** Subnetwork identification
  - **7.2** Subnetwork visualisation
  - **7.3** Gene nodes in the subnetwork
  - **7.4** Edges in the subnetwork
- **8** Combinatorial attack analysis
  - **8.1** R function CombAttack
  - **8.2** Individual nodes
  - **8.3** Two-node combination
- **9** R session information
- **10** Flow cytometry data

# Identification of and combinatorial attack on a gene subnetwork active during experimental autoimmune myocarditis

## 8.1 R function CombAttack

We implement an R function called `CombAttack` to calculate attackness for individual nodes and nodes in combination (see the function parameter `combine`) in a network (see the function parameter `ig`).

```
## ig: an igraph object
## combine: a list containing individual nodes or nodes combined for combinatorial attack
CombAttack <- function(ig, combine)
{

    max.comp.orig <- max(igraph::components(ig)$csize)
    n <- vcount(ig)
    
    res <- NULL
    if(!is.null(combine)){
        m <- length(combine)
        max.comp.removed <- rep(max.comp.orig, m)
        nodes.removed <- rep(max.comp.orig, m)
        removed.pct <- rep(max.comp.orig, m)
        pb <- dplyr::progress_estimated(m)
        for(i in seq_len(m)){
            pb$tick()$print()
            ind <- match(V(ig)$name, combine[[i]])
            v <- V(ig)$name[!is.na(ind)]
            nodes.removed[i] <- paste(v,collapse=',')
            g.manual <- igraph::delete_vertices(ig, v)
            max.comp.removed[i] <- max(igraph::components(g.manual)$csize)
            removed.pct[i] <- 1 - igraph::vcount(g.manual) / n
        }
        comp.pct <- max.comp.removed/max.comp.orig
        res <- tibble::tibble(frac.disconnected=1-comp.pct, frac.removed=removed.pct, nodes.removed=nodes.removed)
    }
    
    return(res)
}
```
