# Supplementary material for: Transcriptomic Analysis of Inflammatory Cardiomyopathy Identifies Molecular Signatures of Disease and Informs in silico Prediction of a Network-Based Rationale for Therapy
Source: Front Immunol. 2021 Mar 5;12:640837. doi: 10.3389/fimmu.2021.640837 (PMC7973371; doi:10.3389/fimmu.2021.640837)
Supplement: Supplementary file 2 [file Data_Sheet_2.zip › Myocarditis/subnetwork-identification.html]

7.1 Subnetwork identification | Identification of and combinatorial attack on a gene subnetwork active during experimental autoimmune myocarditis


- Myocarditis
- **1** Overview
- **2** RNAseq analysis (quality control and differential analysis)
- **3** List of differentially expressed genes
- **4** R packages required
- **5** Gene groupings
  - **5.1** R function Upset
  - **5.2** Group visualisation
  - **5.3** Grouped genes
  - **5.4** Heatmap visualisation
- **6** Pathway analysis
  - **6.1** Enrichment analysis
  - **6.2** Enriched pathways
- **7** Subnetwork analysis
  - **7.1** Subnetwork identification
  - **7.2** Subnetwork visualisation
  - **7.3** Gene nodes in the subnetwork
  - **7.4** Edges in the subnetwork
- **8** Combinatorial attack analysis
  - **8.1** R function CombAttack
  - **8.2** Individual nodes
  - **8.3** Two-node combination
- **9** R session information
- **10** Flow cytometry data

# Identification of and combinatorial attack on a gene subnetwork active during experimental autoimmune myocarditis

## 7.1 Subnetwork identification

```
library(XGR)
library(dnet)
RData.location <- "http://galahad.well.ox.ac.uk/Myocarditis"
```

```
ig.KEGGmm.list <- xRDataLoader("ig.KEGGmm.list", RData.location=RData.location)

## the gene interaction network (the parent network) merged from 11 KEGG pathways identified above
vec_pathways <- c("Chemokine signaling pathway", "Cytokine-cytokine receptor interaction", "TNF signaling pathway","Viral protein interaction with cytokine and cytokine receptor", "NF-kappa B signaling pathway","Cell adhesion molecules (CAMs)","NOD-like receptor signaling pathway","Osteoclast differentiation","Natural killer cell mediated cytotoxicity","Complement and coagulation cascades","Antigen processing and presentation")
ind <- match(vec_pathways, names(ig.KEGGmm.list))
ig <- ig.KEGGmm.list[ind] %>% xCombineNet(combineBy='union', attrBy="intersect", verbose=TRUE)
ig <- dnet::dNetInduce(g=ig, nodes_query=V(ig)$name, knn=0, remove.loops=FALSE, largest.comp=TRUE) %>% as.undirected()

## logFC matrix
mat_FC <- read_delim('DE_genes.txt.gz', delim='\t') %>% select(mgi_symbol,time,logFC) %>% group_by(mgi_symbol,time) %>% summarise(logFC=median(logFC)) %>% ungroup() %>% pivot_wider(names_from=time, values_from=logFC) %>% column_to_rownames('mgi_symbol')

## mat_FDR matrix
mat_FDR <- read_delim('DE_genes.txt.gz', delim='\t') %>% select(mgi_symbol,time,FDR) %>% group_by(mgi_symbol,time) %>% summarise(FDR=min(FDR)) %>% ungroup() %>% pivot_wider(names_from=time, values_from=FDR) %>% column_to_rownames('mgi_symbol')

## aggregated FDR across timepoints
aFDR <- dnet::dPvalAggregate(mat_FDR, method="fishers")

## identification of gene subnetwork
subg <- xSubneterGenes(aFDR, network.customised=ig, seed.genes=T, subnet.size=50, verbose=T)
```

Nodes and edges can be output into subnetwork\_node\_info.txt and subnetwork\_edge\_info.txt, respectively.

```
# gene nodes: write into a file 'subnetwork_node_info.txt'
subg %>% igraph::as_data_frame("vertices") %>% as_tibble() %>% select(name,description) %>% inner_join(mat_FC %>% as_tibble(rownames='name'), by='name') %>% inner_join(mat_FDR %>% as_tibble(rownames='name'), by='name') -> df_nodes
colnames(df_nodes) <-  colnames(df_nodes) %>% str_replace_all('.x','.FC') %>% str_replace_all('.y','.FDR')
df_nodes %>% write_delim('subnetwork_node_info.txt', delim='\t')

# interacting edges: write into a file 'subnetwork_edge_info.txt'
subg %>% igraph::as_data_frame("edges") %>% as_tibble() %>% write_delim('subnetwork_edge_info.txt', delim='\t')
```
