# Supplementary material for: Transcriptomic Analysis of Inflammatory Cardiomyopathy Identifies Molecular Signatures of Disease and Informs in silico Prediction of a Network-Based Rationale for Therapy
Source: Front Immunol. 2021 Mar 5;12:640837. doi: 10.3389/fimmu.2021.640837 (PMC7973371; doi:10.3389/fimmu.2021.640837)
Supplement: Supplementary file 2 [file Data_Sheet_2.zip › Myocarditis/enrichment-analysis.html]

6.1 Enrichment analysis | Identification of and combinatorial attack on a gene subnetwork active during experimental autoimmune myocarditis


- Myocarditis
- **1** Overview
- **2** RNAseq analysis (quality control and differential analysis)
- **3** List of differentially expressed genes
- **4** R packages required
- **5** Gene groupings
  - **5.1** R function Upset
  - **5.2** Group visualisation
  - **5.3** Grouped genes
  - **5.4** Heatmap visualisation
- **6** Pathway analysis
  - **6.1** Enrichment analysis
  - **6.2** Enriched pathways
- **7** Subnetwork analysis
  - **7.1** Subnetwork identification
  - **7.2** Subnetwork visualisation
  - **7.3** Gene nodes in the subnetwork
  - **7.4** Edges in the subnetwork
- **8** Combinatorial attack analysis
  - **8.1** R function CombAttack
  - **8.2** Individual nodes
  - **8.3** Two-node combination
- **9** R session information
- **10** Flow cytometry data

# Identification of and combinatorial attack on a gene subnetwork active during experimental autoimmune myocarditis

## 6.1 Enrichment analysis

```
library(XGR)
RData.location <- "http://galahad.well.ox.ac.uk/Myocarditis"
```

```
## define gene groups
list_vec <- df_full %>% group_by(code) %>% summarise(member=list(member)) %>% deframe()
names(list_vec) <- paste0(names(list_vec), ' (', c('LTr','LTi','MTr','MPr','MTi','MPi','EPi'), ')')

## define the test background
background <- read_delim('DE_genes.txt.gz', delim='\t') %>% pull(mgi_symbol) %>% unique()

## define KEGG pathways
ontology.customised <- xRDataLoader("org.Mm.egKEGG", RData.location=RData.location)

## do enrichment analysis
ls_df <- lapply(seq(length(list_vec)), function(i){ 
    eTerm <- xEnricherGenes(data=list_vec[[i]], background=background, ontology.customised=ontology.customised, test="fisher", size.range=c(10,1000), min.overlap=10)
    if(!is.null(eTerm)){
        xEnrichViewer(eTerm, top_num='all', sortBy="adjp", details=T) %>% mutate(group=names(list_vec)[i])
    }
})
df_output <- do.call(rbind, ls_df) %>% filter(namespace %in% c('Environmental Process','Organismal Systems')) %>% as_tibble() %>% dplyr::group_by(group,namespace) %>% dplyr::top_n(5,-pvalue) %>% dplyr::ungroup() %>% filter(adjp < 0.05) %>% select(group,namespace,name,nOverlap,adjp,members_Overlap)

# write into a file 'Enrichment_KEGG.txt'
df_output %>% write_delim('Enrichment_KEGG.txt',delim='\t')
```

Results stored in `df_output`.

```
df_output
## # A tibble: 30 x 6
##    group    namespace   name        nOverlap     adjp members_Overlap           
##    <chr>    <chr>       <chr>          <dbl>    <dbl> <chr>                     
##  1 0-0-0-1… Organismal… Thermogene…       93 7.70e-30 Acsl1, Acsl6, Adcy5, Adcy…
##  2 0-0-0-1… Organismal… Retrograde…       47 1.00e-14 Adcy5, Adcy8, Adcy9, Cacn…
##  3 0-0-0-1… Organismal… Cardiac mu…       31 8.70e-10 Actc1, Asph, Atp1a2, Atp1…
##  4 0-0-0-1… Environmen… Neuroactiv…       27 1.80e- 3 Adra1a, Adra1d, Adra2b, A…
##  5 0-0-0-1… Organismal… PPAR signa…       15 1.50e- 2 Acadl, Acadm, Acsl1, Acsl…
##  6 0-0-0-1… Organismal… Adrenergic…       26 1.60e- 2 Actc1, Adcy5, Adcy8, Adcy…
##  7 0-0-1-0… Environmen… NF-kappa B…       19 2.40e- 4 Bcl2l1, Ddx58, Gadd45b, I…
##  8 0-0-1-0… Environmen… TNF signal…       19 2.70e- 3 Atf6b, Casp3, Casp7, Casp…
##  9 0-0-1-0… Organismal… Osteoclast…       19 5.50e- 3 Camk4, Fosb, Fosl1, Fosl2…
## 10 0-0-1-0… Environmen… MAPK signa…       33 6.20e- 3 Angpt2, Cacna1d, Casp3, C…
## # … with 20 more rows
```
