# Supplementary material for: Transcriptomic Analysis of Inflammatory Cardiomyopathy Identifies Molecular Signatures of Disease and Informs in silico Prediction of a Network-Based Rationale for Therapy
Source: Front Immunol. 2021 Mar 5;12:640837. doi: 10.3389/fimmu.2021.640837 (PMC7973371; doi:10.3389/fimmu.2021.640837)
Supplement: Supplementary file 2 [file Data_Sheet_2.zip › Myocarditis/gene-nodes-in-the-subnetwork.html]

7.3 Gene nodes in the subnetwork | Identification of and combinatorial attack on a gene subnetwork active during experimental autoimmune myocarditis


- Myocarditis
- **1** Overview
- **2** RNAseq analysis (quality control and differential analysis)
- **3** List of differentially expressed genes
- **4** R packages required
- **5** Gene groupings
  - **5.1** R function Upset
  - **5.2** Group visualisation
  - **5.3** Grouped genes
  - **5.4** Heatmap visualisation
- **6** Pathway analysis
  - **6.1** Enrichment analysis
  - **6.2** Enriched pathways
- **7** Subnetwork analysis
  - **7.1** Subnetwork identification
  - **7.2** Subnetwork visualisation
  - **7.3** Gene nodes in the subnetwork
  - **7.4** Edges in the subnetwork
- **8** Combinatorial attack analysis
  - **8.1** R function CombAttack
  - **8.2** Individual nodes
  - **8.3** Two-node combination
- **9** R session information
- **10** Flow cytometry data

# Identification of and combinatorial attack on a gene subnetwork active during experimental autoimmune myocarditis

## 7.3 Gene nodes in the subnetwork

Available at subnetwork\_node\_info.txt or below.
