# Supplementary figures and images for: Transcriptomic Analysis of Inflammatory Cardiomyopathy Identifies Molecular Signatures of Disease and Informs in silico Prediction of a Network-Based Rationale for Therapy
Source: Front Immunol. 2021 Mar 5;12:640837. doi: 10.3389/fimmu.2021.640837 (PMC7973371; doi:10.3389/fimmu.2021.640837)

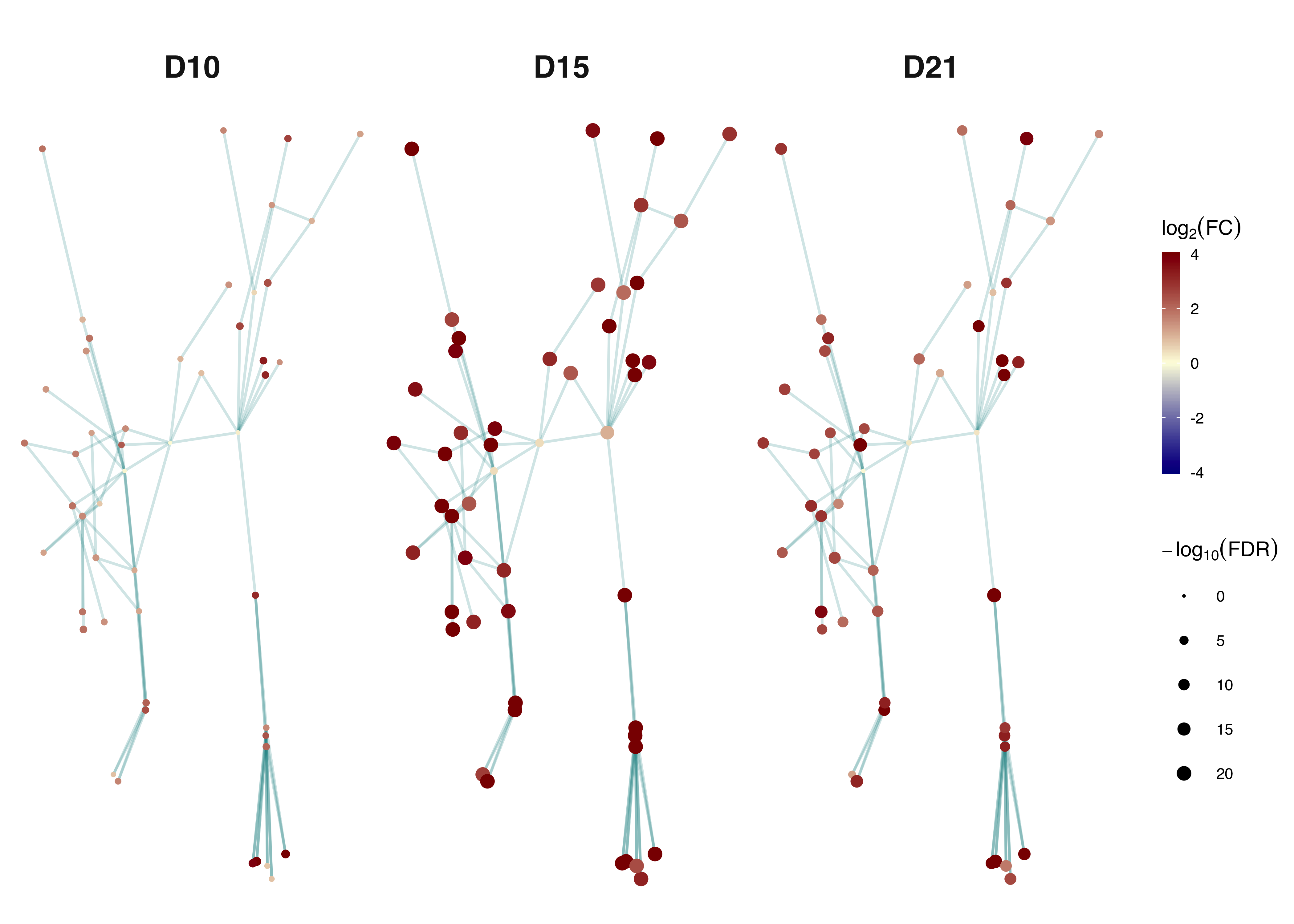

Supplement: Supplementary file 2 [file Data_Sheet_2.zip › Myocarditis/Myocarditis_files/figure-html/unnamed-chunk-20-1.png]

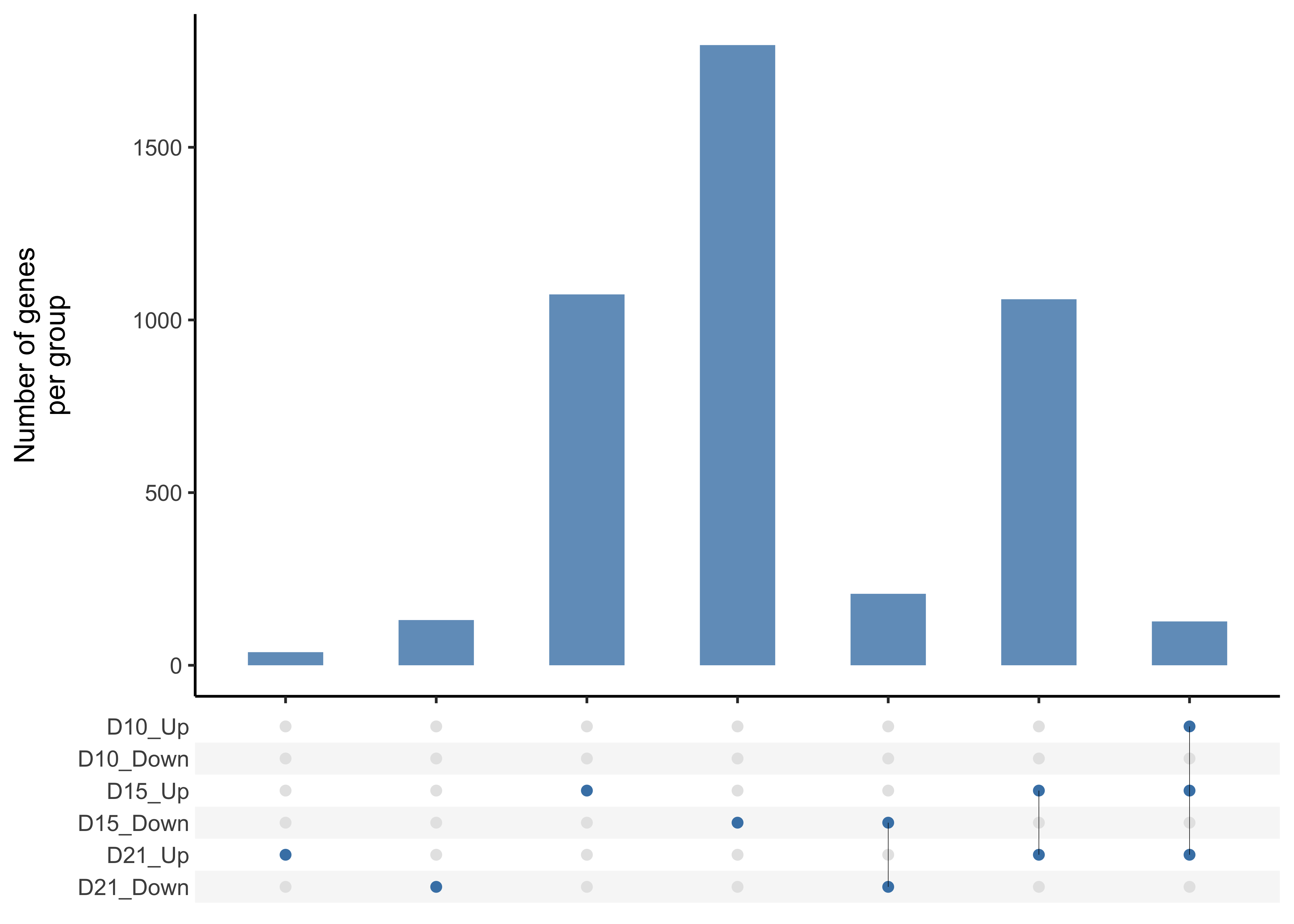

Supplement: Supplementary file 2 [file Data_Sheet_2.zip › Myocarditis/Myocarditis_files/figure-html/unnamed-chunk-8-1.png]

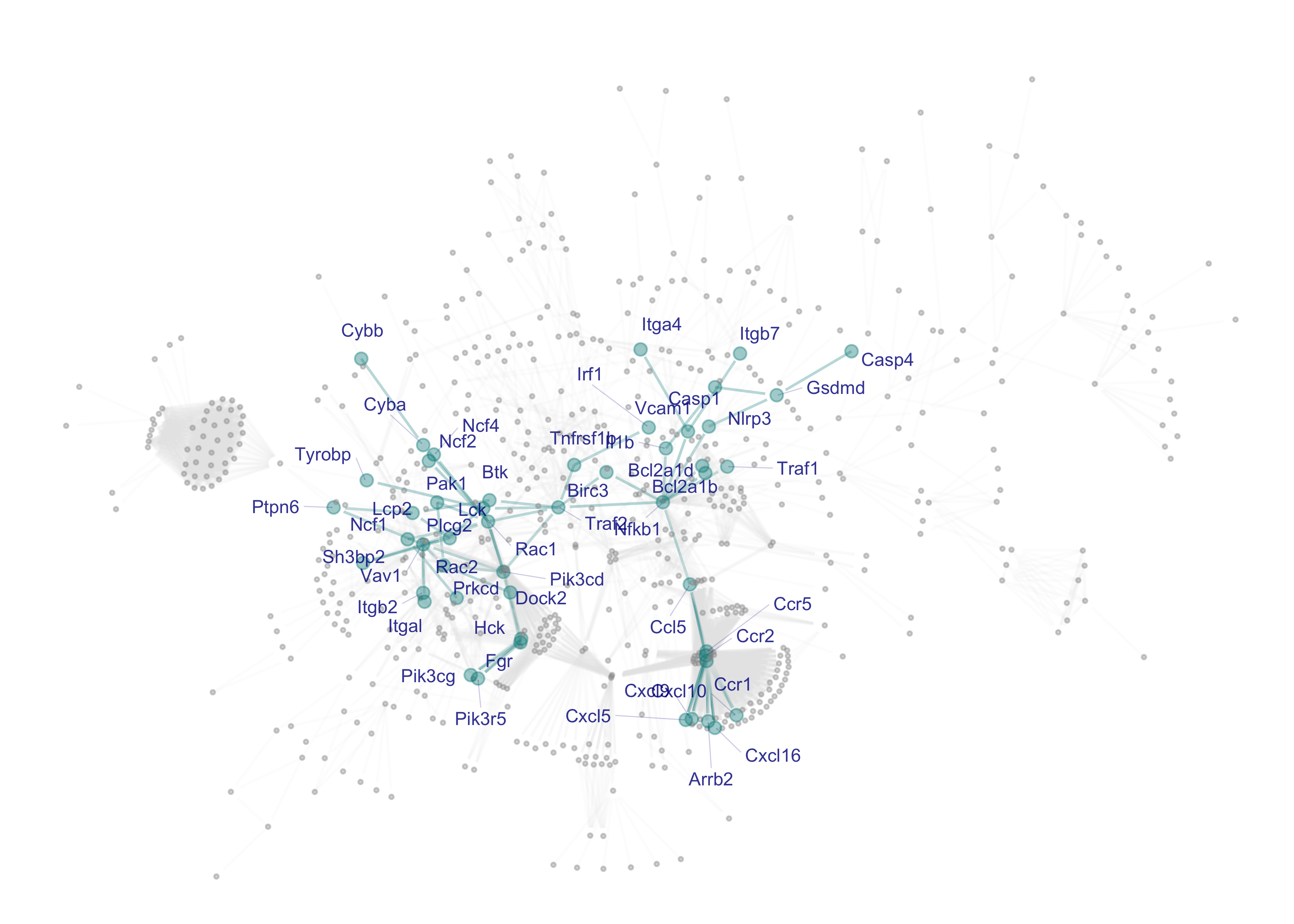

Supplement: Supplementary file 2 [file Data_Sheet_2.zip › Myocarditis/Myocarditis_files/figure-html/unnamed-chunk-21-1.png]

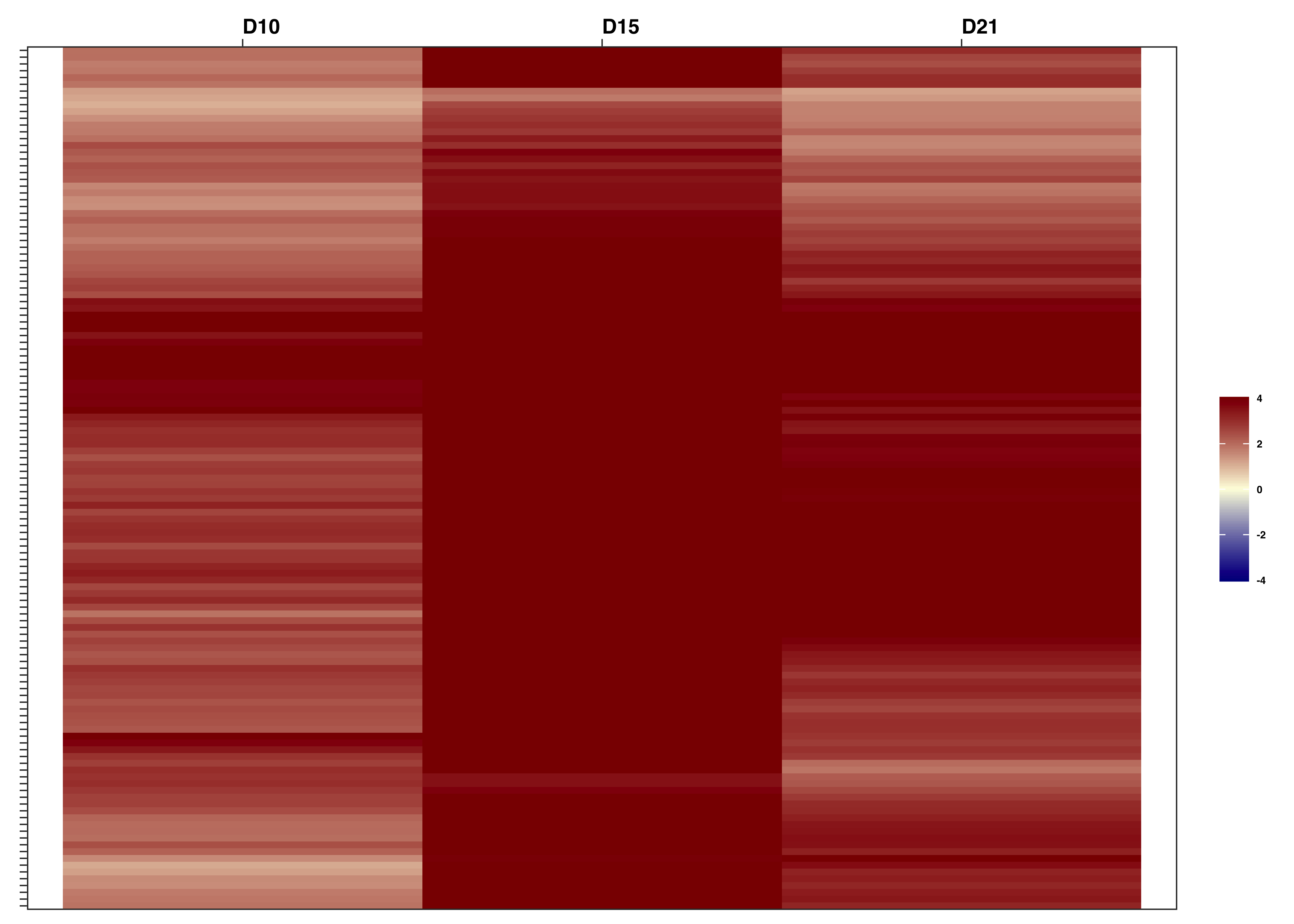

Supplement: Supplementary file 2 [file Data_Sheet_2.zip › Myocarditis/Myocarditis_files/figure-html/unnamed-chunk-10-1.png]
